# The quality of care delivered to residents in long-term care in Australia: an indicator-based review of resident records (CareTrack Aged study)

**DOI:** 10.1186/s12916-023-03224-8

**Published:** 2024-01-23

**Authors:** Peter D. Hibbert, Charlotte J. Molloy, Ian D. Cameron, Leonard C. Gray, Richard L. Reed, Louise K. Wiles, Johanna Westbrook, Gaston Arnolda, Rebecca Bilton, Ruby Ash, Andrew Georgiou, Alison Kitson, Clifford F. Hughes, Susan J. Gordon, Rebecca J. Mitchell, Frances Rapport, Carole Estabrooks, Gregory L. Alexander, Charles Vincent, Adrian Edwards, Andrew Carson-Stevens, Cordula Wagner, Brendan McCormack, Jeffrey Braithwaite

**Affiliations:** 1 Australian Institute of Health Innovation, Macquarie University, 75 Talavera Rd, North Ryde, Sydney, NSW 2109 Australia; 2 IIMPACT in Health, Allied Health and Human Performance, University of South Australia, North Terrace, Adelaide, SA 5000 Australia; 3 South Australian Health and Medical Research Institute, North Terrace, Adelaide, SA 5000 Australia; 4 John Walsh Centre for Rehabilitation Research, Northern Sydney Local Health District, Faculty of Medicine and Health, University of Sydney, Kolling Institute, Reserve Rd, St Leonards, NSW 2065 Australia; 5 Centre for Health Services Research, Faculty of Medicine, The University of Queensland, Princess Alexandra Hospital Campus, Woolloongabba, QLD 4102 Australia; 6 Discipline of General Practice, College of Medicine and Public Health, Flinders University, Sturt Rd, Bedford Park, SA 5042 Australia; 7 Caring Futures Institute, College of Nursing and Health Sciences, Flinders University, Sturt Rd, Bedford Park, SA 5042 Australia; 8 Faculty of Nursing, University of Alberta, Edmonton Clinic Health Academy, 11405-87 Avenue, Edmonton, AB T6G 1C9 Canada; 9 Columbia University School of Nursing, 560 W. 168Th, New York, NY USA; 10 Department of Experimental Psychology, Radcliffe Observatory, University of Oxford, Woodstock Road, Oxford, OX2 6GG England, UK; 11 PRIME Centre Wales & Division of Population Medicine, Cardiff University, 8Th Floor Neuadd Meirionnydd, Heath Park, Cardiff, Wales CF14 4YS UK; 12 Netherlands Institute for Health Services Research, Otterstraat 118, Utrecht, 3513 CR The Netherlands; 13 Amsterdam University Medical Center/VU University, Van Der Boechorststraat 7, 1081 HV Amsterdam, The Netherlands; 14 The Susan Wakil School of Nursing and Midwifery, Faculty of Medicine and Health, University of Sydney, City Road, Sydney, NSW 2006 Australia

**Keywords:** Quality of care, Aged care, Evidence-based care, Long-term care, Clinical audit, Healthcare quality indicators, Healthcare evidence-based management, Guideline adherence

## Abstract

**Background:**

This study estimated the prevalence of evidence-based care received by a population-based sample of Australian residents in long-term care (LTC) aged ≥ 65 years in 2021, measured by adherence to clinical practice guideline (CPG) recommendations.

**Methods:**

Sixteen conditions/processes of care amendable to estimating evidence-based care at a population level were identified from prevalence data and CPGs. Candidate recommendations (*n* = 5609) were extracted from 139 CPGs which were converted to indicators. National experts in each condition rated the indicators via the RAND-UCLA Delphi process. For the 16 conditions, 236 evidence-based care indicators were ratified.

A multi-stage sampling of LTC facilities and residents was undertaken. Trained aged-care nurses then undertook manual structured record reviews of care delivered between 1 March and 31 May 2021 (our record review period) to assess adherence with the indicators.

**Results:**

Care received by 294 residents with 27,585 care encounters in 25 LTC facilities was evaluated. Residents received care for one to thirteen separate clinical conditions/processes of care (median = 10, mean = 9.7). Adherence to evidence-based care indicators was estimated at 53.2% (95% CI: 48.6, 57.7) ranging from a high of 81.3% (95% CI: 75.6, 86.3) for Bladder and Bowel to a low of 12.2% (95% CI: 1.6, 36.8) for Depression. Six conditions (skin integrity, end-of-life care, infection, sleep, medication, and depression) had less than 50% adherence with indicators.

**Conclusions:**

This is the first study of adherence to evidence-based care for people in LTC using multiple conditions and a standardised method. Vulnerable older people are not receiving evidence-based care for many physical problems, nor care to support their mental health nor for end-of-life care. The six conditions in which adherence with indicators was less than 50% could be the focus of improvement efforts.

**Supplementary Information:**

The online version contains supplementary material available at 10.1186/s12916-023-03224-8.

## Background

Relatively little is known about the level of evidence-based care provided to older adults living in long-term care (LTC) at a population level. Knowledge of evidence-based care in this sector is limited to single conditions such as diabetes, or a limited set of indicators, or studied in a small number of sites [1–3]. Unlike healthcare, population-based LTC studies using a standardised method across multiple conditions/processes of care have not been undertaken.

The reliable delivery of evidence-based care to LTC residents is a fundamental human right and is important to maximise their quality of life and reduce the incidence of adverse events. For example, the prevalence and chronicity of pain among LTC residents is under-detected and pain is therefore often inadequately managed [4]. As a result, residents can experience reduced quality of life with impaired physical and cognitive functioning, poor emotional and mental well-being, and increased social isolation [5]. Polypharmacy (> 9 concurrent medications) and overuse of specific agents such as antipsychotics and opiates are common in LTCs and can increase the risk of adverse events including cerebrovascular accidents, cognitive deterioration, and falls [6]. National reports into LTC in Australia [7–9], the United States (US) [10], the United Kingdom (UK) [11, 12] and Canada [13] repeatedly highlight major safety and quality issues for residents including neglect of wounds, incontinence, failure to recognise malnutrition, and poor management of medication which can be, in part, related to evidence-based care not being delivered to residents in a reliable manner.

Providing evidence-based care to elderly residents in LTC is likely to become more challenging. Populations in high-income countries are ageing, and worldwide, the number of persons aged ≥ 80 years is expected to triple between 2020 and 2050, reaching 426 million [14]. More elderly residents are presenting with co- and multi-morbidities [15], fragility and cognitive decline [16]. Scarcity of financial resources and appropriately trained staff and a rapidly changing evidence-base provide further stress to LTC systems [16]. Given these sustainability challenges for LTC, understanding the level of evidence-based care delivered to this vulnerable population, now and into the future, to help direct local and system-level quality improvement initiatives, is vital.

The aim of this study, CareTrack Aged, was to estimate the prevalence of evidence-based care, as measured by adherence to clinical practice guideline (CPG) recommendations in the care received by a population-based sample of Australian residents in LTC aged ≥ 65 years in 2021.

## Methods

The CareTrack Aged study methods have been published elsewhere [17, 18]. We reviewed a sample of 294 care records of LTC residents aged ≥ 65 years as of March 1st 2021, against indicators derived from CPG recommendations for care delivered between 1 March 2021 and 31 May 2021 (our record review period).

### Development and ratification of clinical indicators

**Fig. 1:**
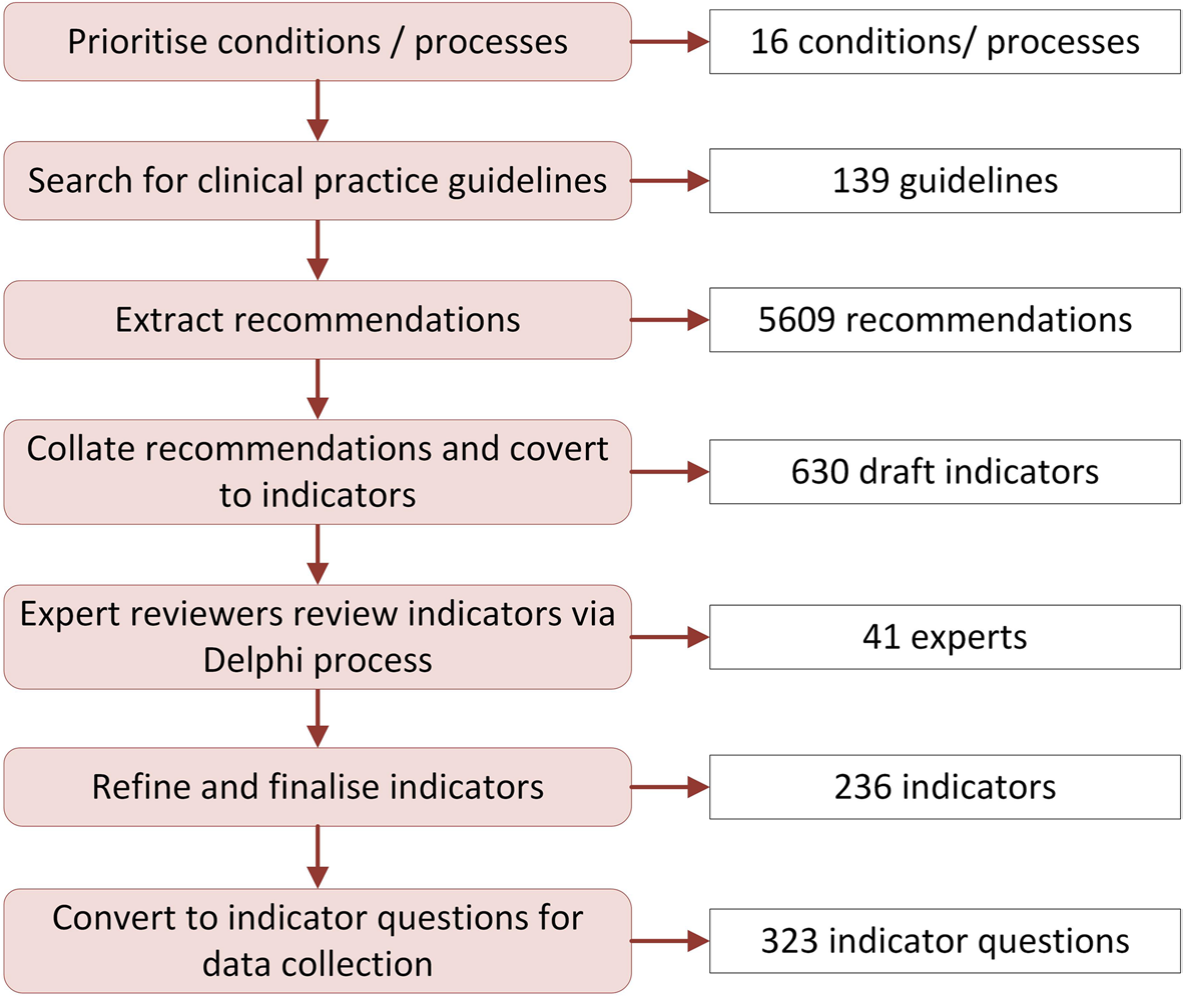
The process for developing and ratifying CareTrack Aged evidence-based care indicators following Hibbert et al. (2022) [18]

We aimed to develop a set of indicators that represented evidence-based care delivered to residents of Australian LTC facilities in 2021. The RAND-UCLA Delphi method to develop indicators was applied [18, 19] (Fig. 1). Sixteen medical conditions or processes of care (Table 1) were selected for inclusion based on a systematic international search for prevalence and burden of disease data, CPGs, and indicator sets relevant to LTC published between 2013 and 2018 [17, 18]. These included high prevalence conditions, such as cognitive impairment which affects over half (54%) of LTC residents [20], and frequently used processes of care, such as medication management [21].

**Table 1:** Examples of included indicators by phase of care and quality type

Condition/process of care	Example indicator	CareTrack Aged indicator number	Phase of care	Quality type^a^
Admission	Residents on admission had a skin wound risk assessment	ADMI17	Diagnosis/assessment	Underuse
Bladder and bowel	Residents with chronic constipation had an individualised bowel management plan	BLBO18	Treatment	Underuse
Cognitive impairment	Residents with delirium or dementia who take anticholinergic medications had their medications reviewed monthly	COGI20	Monitoring/review	Underuse
Depression	Residents who have depression had a comprehensive multidisciplinary care plan	DEPR04	Treatment	Underuse
Dysphagia	Residents who had a choking incident were monitored for 3 days for swallowing difficulties	DYSP07	Monitoring/review	Underuse
End-of-life care	Residents who are dying were prescribed anticipatory medicines with documentation of indications for use, and a range of doses and routes of administration	EOLC20	Treatment	Underuse
Hearing and vision	Residents presenting for the first time with hearing difficulties had an otoscopic examination to exclude impacted wax and acute infection	HEVI01	Diagnosis/assessment	Underuse
Infection	Residents who have asymptomatic bacteriuria or asymptomatic pyuria received antibiotic treatment	INFC18	Treatment	Overuse
Medication	Residents prescribed benzodiazepines OR antipsychotics had a written tapering plan	MEDI08	Treatment	Underuse
Mobility and falls	Residents at medium/high risk of falling received a multifactorial intervention	MOBI05	Treatment	Underuse
Nutrition and hydration	Residents received monthly screening for malnutrition using a validated tool	ADMI22	Diagnosis/assessment	Underuse
Oral and dental care	Residents who have a change in the condition of their mouth or teeth had an oral health assessment	ORAL09	Diagnosis/assessment	Underuse
Pain	Residents who have acute pain received long-acting opioid preparations for pain management	PAIN27	Treatment	Overuse
Restraint	Residents who are being physically restrained had a cognitive assessment prior to restraint use	REST01	Diagnosis/assessment	Underuse
Skin integrity	Residents who have a pressure injury were repositioned at least every 4 h	SKIN19	Treatment	Underuse
Sleep	Residents who have insomnia and are prescribed pharmacological interventions were monitored	SLEP06	Monitoring/review	Underuse

^a^*Underuse* — actions which are recommended, but not undertaken; *Overuse* — actions which are either not indicated or contraindicated (e.g. unjustified antibiotic prescription, or diagnostic testing)

Recommendations (*n* = 5609) were extracted from 139 CPGs relevant to the 16 conditions/processes of care and screened for eligibility; the research team excluded 2136 recommendations by consensus for one or more of four reasons: (1) weak strength of the recommendation indicated by wording such as “may” or “could”; (2) low likelihood of the information being documented; (3) guiding statements without recommended actions (e.g. “consideration should be given to”); and (4) “structure-level” recommendations (e.g. general instructions for personal protective equipment) [18, 22]. The 3473 remaining recommendations were grouped into a standardised indicator format and, after consolidation of similar recommendations, 1790 were used to draft 630 initial indicators [18].

Australian-based LTC experts (*n* = 41) were recruited to review the draft indicators [18]. Their profiles are outlined in Additional file 1: Table S1 [18]. Experts ratified the proposed indicators over a two-stage modified Delphi process, working independently to minimise group influence [23].

Experts scored the appropriateness of each of the draft indicators on a 9-point Likert scale (9 = highly appropriate, 1 = not at all appropriate) in line with the RAND-UCLA Delphi method [19]. In addition, they scored the indicators against three more specific criteria (acceptability, feasibility and impact, scored as ‘Yes’/’No’ or ‘Not Applicable’) [18] consistent with the process used in two previous CareTrack studies measuring evidence-based health care delivered to adults [24] and children [25]. Reviewers could also provide additional comments. Feedback was collated to revise indicators between rounds. Indicators with an average appropriateness score of less than 7 or a majority score of ‘No’ across any of the scoring criteria were excluded. This resulted in the removal of 394 indicators leaving 236 representing evidence-based care in LTC residents [18]. These indicators were categorised by the type of quality of care addressed (e.g. underuse, overuse) and type of phases of care (e.g. diagnosis/assessment, treatment, monitoring/review).

A single indicator was frequently separated into multiple indicator questions. For example, one indicator related to residents receiving a comprehensive physical assessment post-fall, within 1 week, of their gait, lower limb muscle strength and joint function. This generated three indicator questions, related to assessment of gait, lower limb muscle strength and joint function. The 236 indicators generated 323 indicator questions that were grouped into 16 conditions/processes of care to assess evidence-based care [18]. Examples of indicators are shown in Table 1, with full listing in Additional file 2: Table S2 [26–129].

### Sampling process

A multistage sampling process was applied. Sampling was initially planned within three Australian states, Queensland, New South Wales and South Australia (SA). However, due to constantly changing government restrictions during the COVID-19 pandemic, only LTC facilities in SA were recruited. The profile of the facilities and residents in SA is similar to Australia (Tables 2 and 3) [130, 131].

**Table 2:** Demographic characteristics of the participants in the study compared to Australian long-term care residents

Characteristic	Study sample (*n* = 294) *n* (%)	South Australian long-term care population^a^ (*n* = 16,751) *n* (%)	Australian long-term care population^a^ (*n* = 187,043) *n* (%)
*Age*			
65–69	9 (3.1)	518 (3.1)	6569 (3.5)
70–74	19 (6.5)	1157 (6.9)	13,726 (7.3)
75–79	27 (9.2)	1807 (10.8)	21,281 (11.4)
80–84	45 (15.3)	2908 (17.4)	33,805 (18.1)
85–89	90 (30.6)	3976 (23.7)	45,352 (24.2)
90–94	65 (22.1)	4137 (24.7)	44,019 (23.5)
95–99	33 (11.2)	1923 (11.5)	19,173 (10.3)
100 +	6 (2.0)	325 (1.9)	3118 (1.7)
*Sex*			
Female	187 (63.6)	11,360 (67.8)	124,143 (66.4)
Male	107 (36.4)	5391 (32.2)	62,900 (33.6)

^a^Source: Australian Institute of Health and Welfare. GEN data: people using aged care Canberra: AIHW; 2021 [130]

**Table 3:** Demographic characteristics of the facilities included in the study compared to Australian long-term care facilities

Characteristic	Study sample (*n* = 25) *n* (%)	SA long-term care population^a^ (*n* = 241) *n* (%)	Australian long-term care population^a^ (*n* = 2705) *n* (%)
*Remoteness*			
Major cities of Australia	17 (68)	157 (65)	1695 (63)
Inner regional Australia	7 (28)	40 (17)	650 (24)
Outer regional Australia	1 (4)	42 (17)	318 (12)
Remote Australia	0 (0)	2 (1)	32 (1)
Very remote Australia	0 (0)	0 (0)	10 (< 1)
*Organisation type*			
Charitable	3 (12)	45 (19)	515 (19)
Community based	5 (20)	40 (17)	412 (15)
Local government	1 (4)	2 (1)	25 (1)
Private incorporated body	0 (0)^b^	68 (28)	931 (28)
Publicly listed company	0 (0)	0 (0)	2 (< 1)
Religious	13 (52)	62 (26)	611 (23)
Religious/charitable	0 (0)	0 (0)	1 (< 1)
State government	3 (12)	24 (10)	208 (8)

^a^South Australian and Australian long-term residential populations are as reported for the Australian Aged Care Service List – Australia as at 30 June 2021 [131]

^b^Only one small facility sampled; considered insufficient to be confident of representativeness so private facilities were removed from the sampling frame

The sampling frame for LTC facilities was the Aged Care Service List [131], which groups LTC facilities into Aged Care Planning Regions [132]. The list includes the number of licensed beds at each facility, the Australian Standard Geographical Classification of Remoteness Areas (Major Cities, Inner Regional, Outer Regional, Remote and Very Remote) and organisation type (Charitable, Community-based, Local Government, Private, Religious, and State Government).

Within facilities, sampling was restricted to permanent residents aged ≥ 65 years on the 1st March 2021 who resided in the facility in the record review period. This period was selected because, in SA, COVID-19 prevalence and associated social restrictions were relatively low.

We aimed to sample the records of 12 residents per facility. We purposively sampled 4 residents each for those admitted within our timeframe (‘admission’) and those who died in our timeframe (‘end-of-life’). Within each consented facility, the eligible residents were identified by the facility and listed in random order; care records were accessed until a quota of 12 was reached.

### Recruitment of long-term care facilities

Within SA, as of 30 June 2021, there were 272 listed facilities operated by 94 separate providers, with 18,847 funded residential beds. Of these, the initial sampling frame was created by restricting to services with the ‘Residential’ Care Type (i.e. excluding ‘Multi-purpose’ and ‘National ATSI Aged Care program’ services), to focus on LTC beds in services with 20 or more funded residential beds, for logistical reasons. The initial sampling frame thus comprised 84 providers (89% of total SA providers), operating 235 facilities (86% of SA) with 18,055 beds (96% of SA) (see Additional file 3: Fig. S1).

For practical reasons, reflecting management realities during the COVID-19 pandemic, facilities beyond a 3-h drive from the SA state capital city (Adelaide) were excluded as were facilities run by private sector organisations, which were proving difficult to recruit. The final sampling frame was thus reduced to 54 providers (64% of the initial sampling frame) operating 150 services (64%) containing 11,345 funded residential beds (63%).

Twenty-four of the 54 providers (44%) were approached directly after being recommended by colleagues or other providers. Of those, thirteen providers (54%) agreed to participate. For 10 providers, all LTC facilities were included (n = 15 facilities) while for the other three a subset of facilities (*n* = 10 from a total of 27 eligible) were randomly sampled by the providers; sampled facilities included 1927 of 3233 residential beds (59.6%) operated by these three providers.

### Sample considerations

As noted previously, there are 323 *indicator questions*, grouped into 16 conditions/processes of care. Not all indicator questions are assessable for all residents. The underlying unit of analysis is the assessed indicator. As some questions were anticipated to apply to few people, sample sizes were estimated on the basis of that required to achieve a desired precision when assessed indicators are aggregated at the level of the condition/process of care rather than the individual indicator question. For example, in a condition with 10 questions and an average of 56 assessed indicators per question, there would be 560 assessed indicators for analysis of adherence at the level of the condition.

With simple random sampling, approximately 400 assessed indicators are required to obtain estimates with a precision of + / − 5% at an estimated 50% adherence (i.e. the adherence rate that generates the widest binomial confidence interval). We anticipated requiring substantially more than 400 assessed indicator questions per condition to compensate for the ‘design effects’ described below, and have therefore deliberately sought to achieve more than 400 assessed indicator questions per condition. This is not however precise as we do not know, a priori, how many indicator questions will be assessable per resident.

For each selected resident, record reviews were conducted for all conditions/processes relevant to their care, and surveyors determined if each indicator was relevant and, if deemed relevant, determined whether or not care was adherent. Multiple assessed indicators are clustered within a resident’s record and these residents are in turn clustered within a service which are in turn clustered within an organisation. If adherence rates are more similar within than between these clusters, this leads to a design effect, resulting in wider confidence intervals [133]. Estimation of this design effect requires knowledge of the prevalence of the indicator questions, and the intracluster correlation coefficient (ICC), a measure of the extent of within-cluster similarity; neither of these critical pieces of information was available when the study commenced.

Resources had been provided for reviewing 400 care records. As each condition/process contains multiple indicators, each record review can be expected to generate multiple assessed indicators. We therefore generated a set of simulations to assess the implications of clustering by resident, facility and provider on required sample sizes; using the ultimate cluster assumption, the final analysis would adjust for clustering by provider. With resident as the only unit of clustering, any ICC could be tolerated as long as 400 residents were sampled. With facility as the level of clustering, 25 facilities and 80 or more condition-questions assessed per facility, an ICC of 0.05 could be tolerated; with 40 or fewer questions, an ICC of 0.03 could be tolerated. Assuming two facilities per provider, an ICC of 0.02 could be tolerated if 120 or more indicator questions were assessed for the condition; if higher ICCs were encountered, this would result in wider confidence intervals, and vice versa. Based on these simulations it was decided to sample 16 residents in each of 25 facilities (i.e. *n* = 400 care records in total) which was assumed to generate over 1500 assessed indicators for most conditions/processes of care (i.e. 120/provider or 60/facility). This target number of care records was subsequently reduced to 300 residents in total, as a result of restrictions in response to the COVID-19 pandemic. It was decided to retain the number of facilities but reduce the number of residents/facility to 12, allowing tolerance for a higher ICC.

### Data collection tools

A bespoke web-based data collection tool, developed for the CareTrack Australia study [24], was modified for LTC conditions/processes and indicators. A manual (which is available on request) was developed which included instructions, condition/process-specific definitions, inclusion and exclusion criteria, and guidance for assessing indicator eligibility.

### Reviewer engagement, training and agreement based on kappa scores

Three experienced registered LTC nurses were recruited to review care records. They were all employed by the university and were independent of the recruited facilities. Prior to data collection, the reviewers undertook a 1-week training programme. Care records were reviewed on-site at each facility or off-site depending on accessibility of electronic systems: however, all facilities were visited by surveyors to collect data. Surveyors collected the data between October 2021 and September 2022.

Weekly meetings were held between the research team and the surveyors to harmonise surveyors’ views. Mock records were assessed to calculate inter-rater reliability. Among 300 indicators, a substantial level of inter-rater reliability between reviewers was found for indicator eligibility (*k* = 0.71, SD = 0.07) and adherence (*k* = 0.67, SD = 0.06).

### Data collection

Reviewers undertook structured criterion-based care record reviews. One review per eligible condition/process was completed for each resident for the record review period. The reviewers responded to each indicator as ‘Yes’ (care provided during the encounter was consistent with the indicator), ‘No’, or ‘Not Applicable’ (NA; the indicator was not relevant to the encounter because the resident did not meet the inclusion criteria for the indicator). For example, an indicator shown in Table 1, MOBI05, has an inclusion criteria for a particular level of severity (e.g. “medium/high risk of falls”) but this level of severity is not applicable to all residents. If there were multiple instances of a particular indicator (i.e. three falls requiring follow-up in the 3-month period), multiple assessed indicators could be recorded for the same resident.

In a pilot study undertaken in July/August 2021 in two facilities collecting data on 9 residents and 734 eligible indicators, we did not find any residents in the care records who met inclusion criteria for ‘hearing and vision’ and ‘behaviours requiring restraint’. Therefore, we excluded these two conditions from our main data collection as there were no assessed indicators.

### Analysis

The maximum number of indicator questions assessable in each condition/process of care reviewed ranged from six for ‘sleep’, to 40 for ‘pain’ [18] (Additional file 2: Table S2), with the number of eligible responses varying depending on age, sex, relevance and clinical criteria. Overall adherence, adherence per condition/process and adherence at any other aggregate level were estimated as the self-weighted average of the constituent indicator questions for which they were assessed. Each resident was allocated a weight indicative of our best estimate of the number of people they represented in the study population; that weight was applied to each indicator question for which they were assessed. More information on weighting is presented in Additional file 4 [134].

Indicators were clustered within residents who were in turn clustered within facilities which were in turn clustered within providers. Analysis was undertaken in SAS v9.4, using the SURVEYFREQ procedure to control for clustering at the provider level (for all analyses above the indicator level, except analysis of adherence by facility), as the ultimate cluster, weighted to address selective over-sampling. The overall estimate, the estimate by phase of care and the estimate by indicator type (overuse/underuse) were all stratified by both condition and organisation type, the latter aggregated as three pseudo-strata (community, religious, other [charitable, local government or state government]) to avoid single clusters by strata; condition-specific and indicator-specific estimates were solely stratified by organisation type as a pseudo-stratum. Variance was estimated by Taylor series linearization and exact two-side 95% confidence intervals were calculated using the modified Clopper-Pearson method.

For the purposes of informing future research, we estimated the ICCs calculable from our data. We used a well-described method of deriving the ICC for binary responses using the random intercept from the generalised linear mixed model [135]; this was operationalised using PROC GLIMMIX, with the estimate calculated using the Laplace method. ICCs were estimated at the level of the ultimate cluster, the provider.

For individual indicator questions, confidence intervals would be tighter for common conditions and wider for rarer ones. With 25 assessed indicator questions, a confidence interval around estimated adherence of 50% would have a precision of + / − 20% even without adjustment for design effects; it was decided that estimated adherence would not be reported for indicator questions with fewer than 25 assessments.

## Results

### Characteristics of sampled long-term care residents

Of the 300 residents included from 25 facilities, six were removed; three were admitted and died during the target period and three were found to be aged < 65 years. Of the 294 included residents, 73 were admitted during the target period (24.8% of the sample), 61 died during the record review period (20.7% of the sample) and the remaining 159 were residents throughout the record review period (54.4% of sample).

The 294 residents received assessable care (i.e. one or more assessed indicator) for one to thirteen separate clinical conditions/processes of care (median = 10 [IQR: 9–11], mean = 9.7 [SD: 2.53]) and had 23 to 221 assessed indicators (median = 85 [IQR: 61–120], mean = 90.9 [SD: 38.9]). Table 2 compares the age composition of this study population to all Australian and SA LTC residents [130]. Characteristics of the included facilities compared to Australian and SA facilities are shown in Table 3.

### Quality of care indicators

Of 69,454 potentially assessable indicator questions, 41,021 (61%) were designated as not applicable. This left 26,731 assessed indicator questions. Mean prevalence of adherence with evidence-based care indicators, by clinical condition/process, is shown in Table 4. Estimated adherence ranged from 12.2% (95% CI: 1.6, 36.8) for depression to 81.3% (95% CI: 75.6, 86.3) for bladder and bowel. Overall, quality of care was estimated to be adherent for 53.2% (95% CI: 48.6, 57.7) of indicators. Facility-level adherence ranged from 34.1 to 66.4%.

**Table 4:** Evidence-based care by condition/process of care and phase of care in Australian long-term care residents, 2021

Condition/phase of care	*N* residents assessed	*N* assessed indicator questions	% adherence (95% CI)
*By condition/process of care*			
Bladder and bowel	259	1457	81.3 (75.6, 86.3)
Cognitive impairment	197	2766	74.5 (64.9, 82.5)
Oral health	263	517	65.0 (52.7, 76.0)
Dysphagia	95	218	57.0 (37.7, 74.8)
Admission	63	1667	54.3 (49.8, 58.6)
Pain	224	4899	54.0 (40.3, 67.3)
Mobility and falls	270	3790	53.7 (44.1, 63.1)
Nutrition and hydration	273	2388	51.4 (49.4, 53.5)
Skin Integrity	281	2584	49.6 (46.0, 53.3)
End-of-life care	278	2420	44.1 (36.5, 51.9)
Infection	225	2546	34.9 (32.0, 38.0)
Sleep	35	93	33.2 (19.9, 48.8)
Medication	234	1041	26.6 (22.6, 30.9)
Depression	149	345	12.2 (1.6, 36.8)
*By phase of care*			
Referral/consultation	205	567	65.3 (53.2, 76.2)
Treatment	282	10,045	61.6 (54.3, 68.5)
Documentation	72	87	55.7 (36.3, 73.9)
Diagnosis/assessment	294	11,800	51.5 (41.5, 57.5)
Resident/family engagement	57	114	48.4 (32.7, 64.4)
Monitoring/review	282	3019	41.8 (35.0, 48.9)
Information provision	271	1099	10.2 (6.1, 15.8)

Mean adherence was also calculated by the selected phase of care. Estimated adherence was 51.5% (95%C CI: 41.5, 57.5) for diagnosis/assessment, 61.6% (95% CI: 54.3, 68.5) for treatment and 41.8% (95% CI: 35.0, 48.9) for monitoring/review processes (Table 4). Indicators designed to guard against overuse had an estimated adherence of 91.6% (95% CI: 79.6, 97.7), while those signalling care that is necessary (underuse) had an estimated adherence of 52.0% (95% CI: 47.6, 56.4).

We estimated the actual ICCs associated with overall adherence and condition-level adherence, at the level of the provider. The actual ICC associated with the overall estimate of adherence was 0.023. The median ICC at the condition level was 0.046 (IQR: 0.029 to 0.103). ICCs for each condition are listed in Additional file 5; these ranged from 0.009 for nutrition and hydration indicators to 0.542 for depression, where six of 14 providers had 0% adherence, making inter-provider variation a key component of total variation.

A summary of information about indicator questions is presented in Table 5. The number of indicator questions ranged from six for sleep to 40 for pain. The median number of responses to each question ranged from 5 for depression (IQR: 4 to 56) through to 202 for mobility (IQR 190 to 217). Adherence for each indicator with 25 or more assessments is presented in Additional file 2: Table S2. As reported in Table 5, the number of questions with reported adherence ranged from one for sleep to 38 for pain. Within each condition, the median (and the interquartile range) of reported adherences ranged from 10.6% (IQR 0.6 to 17.2%) for the three reported indicator questions for depression, to 92.4% (IQR: 49.4 to 97.1%) for the eight reported bladder and bowel indicator questions.

**Table 5:** Information about indicator questions, by condition/process of care

Condition/process of care	*N* indicator questions with one or more assessed responses	Median number of responses to each question (IQR)	*N* indicator questions with adherence reported^a^	Median reported value for indicator questions with adherence reported^b^ (IQR))
Admission	30	62 (62, 63)	28	55.0 (25.2, 77.9)
Bladder and bowel	20	6 (2, 183)	8	92.4 (49.4, 97.1)
Cognitive impairment	32	78 (24, 146)	24	77.4 (26.1, 94.0)
Depression	11	5 (4, 56)	3	10.6 (0.6, 17.2)
Dysphagia	9	12 (7, 29)	4	61.0 (28.5, 86.1)
End-of-life care	32	55 (53, 57)	29	66.3 (43.0, 84.7)
Infection	32	61 (11, 119)	18	24.3 (4.6, 55.4)
Medication	10	99 (79, 150)	10	24.2 (17.7, 58.9)
Mobility and falls	20	202 (190, 217)	20	42.4 (21.0, 82.8)
Nutrition and hydration	23	76 (16, 112)	17	28.5 (12.0, 62.6)
Oral health	9	21 (12, 22)	2	55.2 (12.4, 98.1)
Pain	40	131 (72, 156)	38	57.6 (23.0, 76.9)
Skin integrity	22	64 (41, 218)	18	48.8 (18.5, 74.8)
Sleep	6	20 (3, 23)	1	18.3 (18.3, 18.3)

^a^Where *n* ≥ 25 responses assessed. Adherence was not reported separately for indicator questions with fewer than this number of assessed responses, due to wide confidence intervals

^**b**^For some questions such as sleep, the median reported differs from the overall weighted average (see Table 4) because a large proportion of assessed indicators were for questions with fewer than 25 assessments, and unreported adherence for these questions was different to that of the reported indicator question(s)

## Discussion

This is the first study of adherence to evidence-based care in LTC facilities at a population level using a standardised method across multiple conditions/processes of care. We found LTC residents received 53.2% of recommended care for 14 conditions/processes. Population-level studies in acute care have similarly found that evidence-based care for adults in the US was 55% [136] and in Australia was 57% [24]. Residents received care for an average of 9.7 assessable conditions, much higher than the studies in acute adult care (e.g. 2.5 in the US [136] and 2.9 in Australia [24]), reflecting the residential nature of LTC and vulnerability of the population. There was considerable variation between conditions/processes, which was also found in the two previous adult healthcare studies [24, 136]. Adherence with indicators for the bladder and bowel condition scored highly with over 80% adherence, and another, cognitive impairment, showed adherence in over 70%. However, for care provided for six conditions (skin integrity, end-of-life care, infection, sleep, medication, and depression) adherence was below 50%.

The results provide valuable insights to identify specific conditions and clinical processes where improvement efforts should be targeted. For example, depression symptoms affect just over half (52%) of all permanent LTC residents [137]. When managing older people with depression, greater vigilance is necessary due to reduced bioavailability [88], risk of drug interactions with polypharmacy [138], and rare side effects such as bone loss [139]. However, we found that only 1% of residents who have depression and who had been receiving antidepressants for 4 weeks were monitored on a monthly basis for side effects (Table S2: Indicator no. DEPR07).

In Australian LTC facilities, approximately 83% of residents die in-house [20, 140]. End-of-life care that people receive in the last months or weeks of their lives should meet their cultural, spiritual, psychosocial and physical needs [141]. Family members who are prepared for a resident’s death through clear communication with LTC staff are less likely to experience complicated grief responses [94, 142]. However, we found less than half (47%) of residents who died in LTC had an individualised care plan including resource needs and involvement of family member needs (EOLC19). On the other hand, 93% of residents who died and who had been in pain were treated with morphine or hydromorphine (EOLC27) and 93% were provided with comfort care measures (EOLC31).

Urinary tract infections (UTIs) are the most common infection treated with antibiotics in Australian LTC facilities [143]. Our results show that 92% of residents who have symptoms of a UTI had a urine sample taken to test for signs of infection within 24 h (INFC16) but only 23.5% of residents with UTI symptoms had a full clinical assessment prior to diagnosis (INFC15). In frail older people, UTIs are more challenging to diagnose [144, 145] and urine sample testing results should not be undertaken in isolation without assessment of the resident’s clinical picture [146]. Relying solely on urine sample testing results contributes to overdiagnosis of UTI and overuse of antibiotics. At a societal level, this contributes to antimicrobial resistance which has been declared by the World Health Organization as one of the top 10 threats facing humanity [147].

The Royal Commission into Aged Care [7], which reported in 2021, is the most contemporary and comprehensive account of why the level of care, including experience, safety, access, and evidence-based care, provided to Australian residents is not meeting societal expectations. At a systems level, these include pressure on government budgets with the LTC sector growing quicker than revenue, poor regulation and systematic monitoring and scrutiny of process measures of care to residents, absence of a consumer voice in the design and delivery of services, and societal assumptions of ageism including within governments and providers. At the level of providers, clinical governance knowledge, skills and investment are markedly under-developed.

A significant contributing factor is the workforce which has changed over the last two decades; nurses comprised about 1:3 of the LTC workforce at the turn of the century and now is 1:4, replaced largely by less skilled personal care workers [7]. In addition, due to poor remuneration, access to medical and allied health skills, including pharmacy, is less than optimal [7]. These structural workforce access issues may explain some of the lower adherence results for conditions that require more specialised knowledge and skills such as end-of-life care, depression, medication and infection control. The key contributing factors relating to workforce found in the Royal Commission align with the most frequently found barriers to delivering evidence-based care in the LTC literature, namely knowledge gaps, organisational support, staff profiles and resources [7, 148].

In terms of the way forward for the LTC sector, as well as addressing the structural deficits such as workforce, the broader health care literature may provide some guidance. Evidence-based overarching strategies such as multi-disciplinary teams, structured handovers and communication [149], embedding co-design with residents, and locally agreed clinical pathways based on evidence should be implemented [150]. The adoption of these strategies in LTC should be underpinned by implementation science principles and skilled local clinical governance teams [150]. At a system and facility level, there should be ongoing routine measurement of evidence-based care [24], not just of common conditions as this study has done, but management of multi-morbidities of residents [15]. Adoption of electronic recording of care can improve both the delivery, via decision support, and efficient measurement of evidence-based care [24].

Our experience of developing indicators for evidence-based care from CPGs for LTC compared to adult [24] and children’s health care [25] was more challenging. CPG guidelines can apply to all adult care, or more specifically to older adults, and even more specifically to LTC. The evidence base for the latter is less well developed and is more likely to include a diverse range of practices, such as routine care, for example, ensuring activities of daily living are reliably undertaken or monitored (Table 1, Dysphagia indicator example, DYSP07) as well as providing complex medical care (Table 1, end-of-life indicator example, EOLC20).

Assessment of the level of evidence-based care provided to LTC residents using care documentation invariably involves clinical judgement by surveyors. In the surveyor manual, during initial training, and the weekly meetings, surveyors were encouraged to apply clinical judgement in the absence of definitions “*to determine what is appropriate and practical*”. Their consistent feedback was that pain was the most difficult condition to assess, in particular, defining new exacerbations. Surveyors also encountered circumstances in the care record when there may be justifiable deviations from evidence-based practices as embodied in the indicators. Similar circumstances were also encountered when residents did not consent to evidence-based care. In these cases, the indicator was scored as adherent.

As to limitations of the study, private facilities could not be recruited and were therefore removed from the sampling frame. There is some evidence that private facilities are likely to have lower adherence to care standards and therefore the prevalence of evidence-based care in Australia is likely to be lower than we have documented [151, 152].

Convenience sampling of facilities may mean that the recruited facilities were not representative of the LTC sector. We collected data from one state, however the profile of the recruited facilities and the residents were similar to those of the whole Australian not-for-profit LTC sector.

There is a potential for self-selection bias. Our provider recruitment rate was 44% which is at the high end of large-scale quality studies (range 8–92%) [25]. If self-selecting facilities were more likely to provide adherent care, this study would have overestimated the quality of care.

The kappa scores were consistent with other care record review studies but, for logistical reasons, were restricted to mock records. This process may have overestimated agreement between reviewers.

The care documented may not reflect the care delivered. All studies seeking to assess the quality of care based on care record review face this possibility. This could work in two ways. Firstly, care delivered is not documented, leading to an underestimation of evidence-based care delivered. This directional bias is well recognised in large-scale quality studies [24, 25, 136]. Secondly, care is not delivered but is documented which would lead to overestimation of evidence-based care. This has been found when checklists are used in healthcare [153]. There have been few studies, particularly recently, of the accuracy of documentation of care records in LTC for the purpose of collecting quality indicators. However, there is a trend that care records overestimate care delivered to residents in pressure ulcers [154], incontinence care [155], feeding assistance [156] and nutritional intake [157]. This may imply that the CareTrack Aged results overestimate the level of evidence-based care delivered to residents.

The indicators were derived from guidelines that were largely published in the years 2013–2018 [18]. As the data review period was 2021, some of the indicators may not have reflected contemporary evidence-based practice [18]. Finally, estimated adherence has wide confidence intervals for almost all indicator questions, and for some conditions/processes of care, especially sleep with only 93 indicator assessments for the six indicator questions. This principally reflects that a small number of indicators were assessed. The width of the confidence intervals suggests that reasonable caution should be exercised when interpreting these indicators. The ICC for overall adherence was in line with that planned for in the sample size estimation, but the vast majority of conditions had ICCs above that which we were able to cater for, leading to wider confidence intervals than desired unless the number of assessed indicators was substantially higher than anticipated. In light of these, future studies should plan to include as large a number of clusters as possible.

## Conclusions

Among a sample of residents in LTC receiving care in Australia in 2021, adherence to evidence-based care indicators for important conditions and processes of care was just over half. Vulnerable older people are not receiving evidence-based care for many physical problems, nor care to support their mental health nor for end-of-life care. The six conditions in which adherence with indicators was less than 50% could be the initial focus of improvement efforts. At a systems level, addressing structural deficits of skills and mix of the workforce, implementing high-reliability practices that we know work, and ongoing measurement of evidence-based practice should be the policy focus.

### Supplementary Information

**Additional file 1: Table S1.** Characteristics of experts reviewing the indicators. Professional group and current primary employer of indicator expert reviewers.

**Additional file 2: Table S2.** CareTrack Aged final clinical indicators and items developed to assess adherence for 16 conditions or processes of care. The clinical indicators and questions are presented by condition, with their source, whether they were measured for under- or over-use, phase of care, number of encounters and adherence (with 95% CI).

**Additional file 3: Figure S1.** Sampling Frame. Sampling Frame flow diagram.

**Additional file 4:** Additional information on sampling and weighting. Additional information on the sampling, weighting and statistical analysis of the data.

**Additional file 5: Table S3.** ICCs overall and by condition. The weighted (unweighted for admission) ICCs overall and by condition.

## Data Availability

Data cannot be shared publicly due to confidentiality and ethical requirements. Requests for data may be sent to the Human Research Ethics Committee of Macquarie University, Ground Floor, 16 Wally’s Walk, Sydney, New South Wales, Australia 2109; Email: ethics.secretariat@mq.edu.au; Telephone: + 61 2 9850 4194, for researchers who meet the criteria for access to confidential data.

## Abbreviations

CPG: Clinical practice guideline
ICC: Intracluster correlation coefficient
LTC: Long-term care
NA: Not applicable
SA: South Australia
UTI: Urinary tract infection
