# Supplementary material for: The quality of care delivered to residents in long-term care in Australia: an indicator-based review of resident records (CareTrack Aged study)
Source: BMC Med. 2024 Jan 23;22:22. doi: 10.1186/s12916-023-03224-8 (PMC10804560; doi:10.1186/s12916-023-03224-8)
Supplement: Supplementary file 1 — Additional file 1: Table S1. Characteristics of experts reviewing the indicators. Professional group and current primary employer of indicator expert reviewers. [file 12916_2023_3224_MOESM1_ESM.docx]

# Additional File 1: Characteristics of experts reviewing the indicators

**TableS1: Characteristics of experts reviewing the indicators^a^**

| **Professional group**^b^ | **n** | **%** |
| --- | --- | --- |
| Nursing | 11 | 23 |
| Research | 8 | 17 |
| Medicine | 7 | 15 |
| Speech pathology | 4 | 9 |
| Optometry | 4 | 9 |
| Dietetics | 2 | 4 |
| Physiotherapy | 2 | 4 |
| Dental | 2 | 4 |
| Pharmacy | 2 | 4 |
| Psychology | 2 | 4 |
| Audiology | 1 | 2 |
| Other | 2 | 4 |
| **Current primary employer^b^** |  |  |
| University | 23 | 53 |
| Aged care health/service provider | 8 | 19 |
| Public health service | 7 | 16 |
| Allied health service provider | 3 | 7 |
| Other | 2 | 5 |

a Re-published with permission: Copyright Clearance Center, Order Number: 5681700653529, Order Date: Dec 03, 2023 from Hibbert et al. Designing clinical indicators for common residential aged care conditions and processes of care: the CareTrack Aged development and validation study International Journal for Quality in Health Care (2022) 34: 2, mzac033

b Experts may be counted more than once if they elected multiple professional groups or primary employers.
