# Supplementary material for: The quality of care delivered to residents in long-term care in Australia: an indicator-based review of resident records (CareTrack Aged study)
Source: BMC Med. 2024 Jan 23;22:22. doi: 10.1186/s12916-023-03224-8 (PMC10804560; doi:10.1186/s12916-023-03224-8)
Supplement: Supplementary file 2 — Additional file 2: Table S2. CareTrack Aged final clinical indicators and items developed to assess adherence for 16 conditions or processes of care. The clinical indicators and questions are presented by condition, with their source, whether they were measured for under- or over-use, phase of care, number of encounters and adherence (with 95% CI). [file 12916_2023_3224_MOESM2_ESM.docx]

# Additional File 2: *CareTrack Aged* final clinical indicators and items developed to assess adherence for 16 conditions or processes of care

The clinical indicators and questions are presented by condition, with their source, whether they were measured for under- or over-use, phase of care, number of encounters and adherence (with 95% CI).

***Table S2: CareTrack Aged* final clinical indicators and items developed to assess adherence for 16 conditions or processes of care**

|  | **Indicator** | **Indicator Question number** | **Indicator Question** | **Source** | **U/O** | **Phase of Care** | **No. of residents assessed** | **No. of indicators assessed** | **Adjusted proportion adherent (95% CI)** |
| --- | --- | --- | --- | --- | --- | --- | --- | --- | --- |
| **Admission** | | | | | | | | | |
| 1 | Residents on admission should have a medical history taken. | ADMI01 | Residents on admission had their medical history taken | (26-28) | U | Diagnosis/ Assessment | 62 | 62 | 94.2% (81.4, 99.2) |
| 2 | Residents on admission should receive a medication history and review. | ADMI02 | Residents on admission had a medication history taken | (26, 28-33) | U | Diagnosis/ Assessment | 62 | 62 | 42.8% (28.5, 58.1) |
| 3 | Residents on admission who have polypharmacy should have renal function calculated. | ADMI03 | Residents on admission who have polypharmacy had renal function calculated | (34) | U | Diagnosis/ Assessment | 50 | 50 | 18.1% (7.5, 34.0) |
| 4 | Residents on admission should have an immunisation status evaluation. | ADMI04 | Residents on admission had an evaluation of their immunisation status | (27) | U | Diagnosis/ Assessment | 63 | 63 | 24.4% (12.8, 39.5) |
| 5 | Residents on admission should receive a medical assessment. | ADMI05 | Residents on admission had a medical assessment | (29) | U | Diagnosis/ Assessment | 62 | 62 | 70.6% (52.7, 84.8) |
| 6 | Residents on admission should receive a cognitive assessment using a standardised tool. | ADMI06 | Residents on admission had a cognitive assessment using a standardised tool | (32) | U | Diagnosis/ Assessment | 63 | 63 | 61.6% (45.7, 75.8) |
| 7 | Residents on admission should receive an end of life needs assessment. | ADMI07 | Residents on admission had an end of life needs assessment | (35-38) | U | Diagnosis/ Assessment | 62 | 62 | 37.4% (22.0, 54.9) |
| 8 | Residents on admission should receive a physical assessment. | ADMI08 | Residents on admission had a physical assessment | (27) (29) (30) (32) | U | Diagnosis/ Assessment | 62 | 62 | 61.8% (45.3, 76.6) |
| 9 | Residents on admission should be asked about the presence of pain. | ADMI09 | Residents on admission were asked about the presence of pain | (39, 40) | U | Diagnosis/ Assessment | 62 | 62 | 95.1% (86.4, 99.0) |
| 10 | Residents on admission should receive a bowel assessment. | ADMI10 | Residents on admission had a bowel assessment | (32) | U | Diagnosis/ Assessment | 62 | 62 | 34.6% (21.1, 50.3) |
| 11 | Residents on admission should receive a urinary continence assessment. | ADMI11 | Residents on admission had a urinary continence assessment | (32) | U | Diagnosis/ Assessment | 62 | 62 | 88.4% (77.7, 95.1) |
| 12 | Residents on admission should receive a functional assessment. | ADMI12 | Residents on admission had a functional assessment | (26, 29, 32, 41) | U | Diagnosis/ Assessment | 63 | 63 | 84.5% (72.4, 92.7) |
| 13 | Residents on admission should receive a neurological assessment. | ADMI13 | Residents on admission had a neurological assessment | (29, 32, 41) | U | Diagnosis/ Assessment | 62 | 62 | 12.3% (4.9, 24.1) |
| 14 | Residents on admission should receive a social/ psychosocial assessment | ADMI14 | Residents on admission had a social/psychosocial assessment | (26, 32, 41) | U | Diagnosis/ Assessment | 63 | 63 | 69.1% (52.1, 83.1) |
| 15 | Residents on admission should have a falls history taken. | ADMI15 | Residents on admission had a falls history taken. | (29, 30) (31, 32, 41) | U | Diagnosis/ Assessment | 62 | 62 | 74.4% (54.8, 88.7) |
| 16 | Residents on admission should be assigned a level of falls risk (e.g., low, medium, high). | ADMI16 | Residents on admission had a level of falls risk assigned to them (e.g., low, medium, high) | (29, 30, 32, 34, 41, 42) | U | Diagnosis/ Assessment | 63 | 63 | 83.7% (72.3, 91.8) |
| 17 | Residents on admission should receive a skin wound risk assessment. | ADMI17 | Residents on admission had a skin wound risk assessment | (43-49) | U | Diagnosis/ Assessment | 63 | 63 | 81.5% (65.0, 92.4) |
| 18 | Residents on admission should receive a hydration status history and assessment. | ADMI18 | Residents on admission had a hydration status history and assessment | (29) | U | Diagnosis/ Assessment | 63 | 63 | 25.9% (12.9, 43.0) |
| 19 | Residents on admission should receive a nutrition/dietary history and assessment. | ADMI19 | Residents on admission had a nutrition/dietary history and assessment | (26, 29, 50) | U | Diagnosis/ Assessment | 63 | 63 | 87.2% (74.2, 95.2) |
| 20 | Residents on admission should be weighed and have their height measured. | ADMI20 | Residents on admission had their weight and height measured | (26, 50) | U | Diagnosis/ Assessment | 60 | 60 | 57.5% (39.8, 73.9) |
| 21 | Residents on admission who are obese should have a comprehensive assessment. | ADMI21 | Residents on admission who are obese had a comprehensive assessment | (51, 52) | U | Diagnosis/ Assessment | 6 | 6 | nc |
| 22 | Residents on admission should receive an assessment for presence of malnutrition using a validated tool. | ADMI22 | Residents on admission had a malnutrition assessment using a validated tool | (50, 53, 54) | U | Diagnosis/ Assessment | 63 | 63 | 49.5% (35.5, 63.5) |
| 23 | Residents on admission who have dysphagia should receive an assessment by a speech pathologist. | ADMI23 | Residents on admission with dysphagia had an assessment by a speech pathologist | (55) | U | Diagnosis/ Assessment | 9 | 9 | nc |
| 24 | Residents on admission should have a general oral health assessment. | ADMI24 | Residents on admission had a general oral health assessment | (26, 56-60) | U | Diagnosis/ Assessment | 63 | 63 | 52.5% (37.6, 67.1) |
| 25 | Residents on admission should be asked whether they have had a dental check up in the preceding 12 months. | ADMI25 | Residents on admission were asked if they had had a dental check-up in the preceding 12 months | (56) | U | Diagnosis/ Assessment | 62 | 62 | 47.6% (32.5, 63.1) |
| 26 | Residents on admission who have not had a dental check up in the preceding 12 months should have a dental check-up. | ADMI26 | Residents on admission who have not had a dental check up in the preceding 12 months had a dental check up | (56, 57) | U | Diagnosis/ Assessment | 29 | 29 | 2.9% (0.0, 16.9) |
| 27 | Residents on admission who have dementia should be assessed for suitability for group activities. | ADMI27 | Residents on admission with dementia were assessed for suitability for group activities | (61) | U | Diagnosis/ Assessment | 40 | 40 | 12.8% (3.0, 31.8) |
| 28 | Residents on admission should have an assessment for the presence of depression using an appropriate tool | ADMI28 | Residents on admission had an assessment for depression using an appropriate tool | (34, 62-65) | U | Diagnosis/ Assessment | 62 | 62 | 9.4% (2.8, 21.7) |
| 29 | Residents on admission who have depression should be assessed for risk of suicide. | ADMI29 | Residents on admission with depression were assessed for risk of suicide | (34, 65, 66) | U | Diagnosis/ Assessment | 37 | 37 | 4.8% (0.5, 17.3) |
| 30 | Residents on admission should have their sleep preferences, patterns and routines documented. | ADMI30 | Residents on admission had their sleep preferences, patterns and routines documented. | (67) | U | Diagnosis/ Assessment | 62 | 62 | 74.0% (59.9, 85.3) |
| **Bladder and Bowel** | | | | | | | | | |
| 1 | Residents who have a significant change in bladder condition should be asked about symptoms of urinary incontinence | BLBO01 | Residents with a significant change in bladder condition were asked about symptoms of urinary incontinence | (68) | U | Diagnosis/ Assessment | 8 | 9 | nc |
| 2 | Residents who present with symptoms of urinary incontinence should have a UI history taken | BLBO02 | Residents who newly presented with symptoms of urinary incontinence had a UI history taken | (34, 68) | U | Diagnosis/ Assessment | 1 | 2 | nc |
| 3 | Residents who present with symptoms of urinary incontinence have a gynaecological/bowel history taken. | BLBO03 | Residents who newly presented with symptoms of urinary incontinence had a gynaecological/ bowel history taken | (34, 68) | U | Diagnosis/ Assessment | 1 | 2 | nc |
| 4 | Residents who newly present with symptoms of urinary incontinence should have a focused physical examination. | BLBO04 | Residents who newly presented with symptoms of urinary incontinence had a focused physical examination | (34, 68) | U | Diagnosis/ Assessment | 1 | 2 | nc |
| 5 | Residents who present with symptoms of urinary incontinence should be assessed with a validated tool | BLBO05 | Residents who newly presented with symptoms of urinary incontinence were assessed with a validated tool | (34, 69) | U | Diagnosis/ Assessment | 1 | 2 | nc |
| 6 | Residents newly diagnosed with urinary incontinence or overactive bladder, should complete a minimum of 3 days of a bladder diary, as part of the initial assessment. | BLBO06 | Residents newly diagnosed with urinary incontinence, or overactive bladder had at least 3 completed days of a bladder diary. | (69, 70) | U | Diagnosis/ Assessment | 1 | 2 | nc |
| 7 | Residents newly diagnosed with or suspected of having urinary incontinence should have urinalysis performed within 24 hours. | BLBO07 | Residents newly diagnosed with or suspected of urinary incontinence had a urinalysis performed within 24 hours | (69, 70) | U | Diagnosis/ Assessment | 1 | 2 | nc |
| 8 | Residents newly diagnosed with urinary incontinence should have it categorised as stress urinary incontinence, mixed urinary incontinence, or urgency urinary incontinence/overactive bladder (OAB) within a week of diagnosis. | BLBO08 | Residents newly diagnosed with urinary incontinence had it categorised as stress urinary incontinence, mixed urinary incontinence, or urgency urinary incontinence/overactive bladder (OAB) within a week of diagnosis. | (70) | U | Diagnosis/ Assessment | 0 | 0 | nc |
| 9 | Residents newly diagnosed with or who have worsening urinary incontinence should have their medications reviewed within 3 days of diagnosis. | BLBO09 | Residents newly diagnosed with or with worsening urinary incontinence had their medications reviewed within 3 days. | (69, 71) | U | Diagnosis/ Assessment | 5 | 5 | nc |
| 10 | Residents who have urinary incontinence should not receive ultrasound unless assessing for residual urine volume. | BLBO10 | Residents with urinary incontinence had an ultrasound for a reason other than assessing for residual urine volume | (70) | O | Diagnosis/ Assessment | 231 | 231 | 100.0% (98.4, 100.0) |
| 11 | Residents who have urinary incontinence should have a current care plan. | BLBO11 | Residents with urinary incontinence had a current care plan | (29, 34) | U | Treatment | 245 | 245 | 98.0% (95.3, 99.3) |
| 12 | Residents^1^ who have urinary incontinence should be informed regarding available treatment options (within 3 days of diagnosis). | BLBO12 | Residents^1^ with urinary incontinence were informed regarding available treatment options within 3 days. | (69) | U | Information provision | 3 | 3 | nc |
| 13 | Residents who have urinary incontinence and are cognitively impaired should receive prompted voiding. | BLBO13 | Residents with urinary incontinence and who were cognitively impaired had prompted voiding. | (69) | U | Treatment | 207 | 207 | 94.2% (87.4, 97.9) |
| 14 | Residents who have a constipation or bowel control problems should receive a bowel assessment | BLBO14 | Residents with constipation or bowel control problems had a bowel assessment | (34, 72-74) | U | Diagnosis/ Assessment | 220 | 222 | 47.9% (34.9, 61.1) |
| 15 | Residents newly diagnosed with faecal incontinence should have an initial management plan (within 3 days). | BLBO15 | Residents newly diagnosed with faecal incontinence had an initial management plan within 3 days | (73) | U | Treatment | 3 | 3 | nc |
| 16 | Residents who are suspected of having constipation should have a physical assessment of the abdomen (within 3 days) | BLBO16 | Residents suspected of having constipation had a physical assessment of the abdomen within 3 days | (74) | U | Diagnosis/ Assessment | 58 | 72 | 6.6% (0.6, 24.4) |
| 17 | Residents who have or are suspected of having constipation should have ongoing monitoring. | BLBO17 | Residents with or who were suspected of having constipation had ongoing monitoring | (74) | U | Monitoring/ Review | 160 | 168 | 96.2% (81.4, 99.9) |
| 18 | Residents who have chronic constipation should receive an individualised bowel management plan. | BLBO18 | Residents with chronic constipation had an individualised bowel management plan | (34, 74) | U | Treatment | 72 | 72 | 50.9% (28.0, 73.5) |
| 19 | Residents who have been identified at risk of constipation, should receive prevention interventions. | BLBO19 | Residents who were identified as at risk of constipation had preventative interventions | (74) | U | Treatment | 197 | 197 | 90.6% (81.1, 96.3) |
| 20 | Residents with recent onset (within the last week) constipation with alarm symptoms should be referred to GP - for further investigation and screening for bowel cancer. | BLBO20 | Residents with recent onset (within the previous week) constipation with alarm symptoms were referred to a GP. | (74) | U | Referral/ Consultation | 4 | 4 | nc |
| 21 | Residents with faecal impaction should be referred for medical review. | BLBO21 | Residents with faecal impaction were referred for medical review. | (74) | U | Referral/ Consultation | 7 | 7 | nc |
| **Cognitive Impairment** | | | | | | | | | |
| 1 | Residents who have symptoms of delirium or dementia, should receive: - a cognitive assessment using a standardised tool AND - medication review AND - physical examination. | COGI01 | Residents with symptoms of delirium or dementia received a cognitive assessment using a standardised tool. | (34, 75-78) | U | Diagnosis/ Assessment | 183 | 183 | 66.1% (51.5, 78.7) |
|  |  | COGI02 | Residents with symptoms of delirium or dementia received a medication review |  | U | Monitoring/ Review | 28 | 35 | 22.3% (5.0, 52.0) |
|  |  | COGI03 | Residents with symptoms of delirium or dementia received a physical examination |  | U | Diagnosis/ Assessment | 27 | 34 | 30.0% (8.3, 61.3) |
| 2 | Residents who present with symptoms of delirium or sudden (over 2 days) changes in baseline, cognition or behaviour should be assessed within 24 hours. | COGI04 | Residents who presented with symptoms of delirium or sudden (over 2 days) changes in baseline cognition or behaviour were assessed within 24 hours. | (75, 79) | U | Diagnosis/ Assessment | 35 | 47 | 61.8% (35.1, 84.0) |
| 3 | Residents who have delirium or are suspected of having delirium should be monitored at least daily using clinical assessments and validated tools. | COGI05 | Residents with delirium or who were suspected of having delirium were monitored at least daily using clinical assessments and validated tools. | (75, 78) | U | Monitoring/ Review | 34 | 44 | 0.0% (0.0, 8.0) |
| 4 | Residents who have delirium should have a current multi-component intervention plan. | COGI06 | Residents with delirium had a current multi-component intervention plan | (78, 79) | U | Treatment | 30 | 34 | 6.0% (0.5, 23.0) |
| 5 | Residents who have delirium and have a multi-component intervention plan had it developed in partnership with the resident (and/or family or substitute decision maker). | COGI07 | Residents with delirium and a multi-component intervention plan had it developed in partnership with the residents (and or the family/substitute decision maker) | (78) | U | Treatment; Resident/ Family engagement | 8 | 11 | nc |
| 6 | Residents who have suspected dementia should have laboratory investigations. | COGI08 | Residents with suspected dementia had laboratory investigations | (80) (34) | U | Diagnosis/ Assessment | 2 | 2 | nc |
| 7 | Residents who have dementia should have a current care and support plan. | COGI09 | Residents with dementia had a current care and support plan | (76-78, 80, 81) | U | Treatment | 172 | 172 | 94.1% (81.8, 99.1) |
| 8 | Residents who have dementia and a care and support plan should have it developed in partnership with the resident and/or family or substitute decision-maker | COGI10 | Residents with dementia and a care a support plan had it developed in partnership with the residents (and or the family/substitute decision maker) | (76-78, 80, 81) | U | Treatment; Resident/ Family engagement | 153 | 153 | 71.9% (57.3, 83.7) |
| 9 | Residents who have dementia should have strategies in place that preserve their abilities and optimise their quality of life (e.g., exercise). | COGI11 | Residents with dementia had strategies in place that preserved their abilities and optimised their quality of life (e.g., exercise). | (76, 78) | U | Treatment | 171 | 171 | 97.0% (92.5, 99.2) |
| 10 | Residents who have dementia without psychosis should not be prescribed anti-psychotics as a first-line approach. | COGI12 | Residents with dementia without psychosis were prescribed anti-psychotics as a first-line approach | (82) | O | Treatment | 108 | 108 | 85.2% (68.8, 95.0) |
| 11 | Residents who have dementia without psychosis should not receive anti-psychotics as a first-line approach. | COGI13 | Residents with dementia without psychosis received anti-psychotics as a first-line approach | (82) | O | Treatment | 105 | 105 | 86.9% (70.1, 96.2) |
| 12 | Residents who are prescribed anti-psychotic medications have the indication documented (delirium with severe agitation and imminent risk of self-harm, or severe psychotic symptoms). | COGI14 | Residents who were prescribed anti-psychotic medications had the indication documented (i.e., delirium with severe agitation and imminent risk of self-harm, or severe psychotic symptoms). | (79) | U | Documentation | 50 | 50 | 53.3% (26.4, 78.9) |
| 13 | Residents^1^ who have dementia should have the benefits and harms of using anti-psychotics discussed with them before starting anti-psychotics. | COGI15 | Residents^1^ with dementia who were prescribed anti-psychotics had the benefits and harms of using anti-psychotics discussed with them prior to prescription. | (77) | U | Information provision; Resident/ Family engagement | 20 | 20 | nc |
| 14 | Residents who have dementia who are prescribed anti-psychotics, were assessed prior to prescribing including: - underlying syndromes, neurological, psychiatric and environmental (interaction) factors - medical state and risk and symptoms. | COGI16 | Residents with dementia who were prescribed anti-psychotics were assessed, prior to prescription, for underlying syndromes, neurological, psychiatric and environmental (interaction) factors | (82) | U | Diagnosis/ Assessment | 19 | 19 | nc |
|  |  | COGI17 | Residents with dementia who were prescribed anti-psychotics were assessed, prior to prescription, for medical state and risk and symptoms; |  | U | Diagnosis/ Assessment | 18 | 19 | nc |
| 15 | Residents who have dementia and are newly prescribed anti-psychotics should be started on the lowest effective dose for the shortest possible time. | COGI18 | Residents with dementia who were newly prescribed anti-psychotics were started on the lowest effective dose for the shortest possible time. | (77, 82) | U | Treatment | 7 | 7 | nc |
| 16 | Residents who have dementia who are using anti-psychotics should be reassessed at least every 6 weeks to determine if they still need anti-psychotics. | COGI19 | Residents with dementia who were using anti-psychotics were reassessed at least every 6 weeks (to determine if antipsychotics still needed) | (77) | U | Monitoring/ Review | 49 | 49 | 16.5% (4.2, 38.5) |
| 17 | Residents who have delirium or dementia and take anticholinergic medications should have their medication reviewed monthly. | COGI20 | Residents with delirium or dementia who take anticholinergic medications had their medications reviewed monthly. | (79, 80) | U | Monitoring/ Review | 26 | 27 | 0.9% (0.0, 14.4) |
| 18 | Residents who have behaviours and psychological symptoms of dementia (BPSD) should have a comprehensive assessment including: - underlying causes AND - analysis of the behaviours AND - physical and mental health AND - level of pain or discomfort AND - side effects of medication AND - the influence of religious and spiritual beliefs and cultural norms AND - physical environmental and interpersonal factors AND - carer(s) health and communication style when interacting with have the resident. | COGI21 | Residents with behaviours and psychological symptoms of dementia (BPSD) had a comprehensive assessment including underlying causes. | (34, 61, 76, 78, 83, 84) | U | Diagnosis/ Assessment | 145 | 145 | 94.8% (87.7, 98.4) |
|  |  | COGI22 | Residents with behaviours and psychological symptoms of dementia (BPSD) had a comprehensive assessment including analysis of the behaviours |  | U | Diagnosis/ Assessment | 146 | 146 | 95.7% (90.3, 98.6) |
|  |  | COGI23 | Residents with behaviours and psychological symptoms of dementia (BPSD) had a comprehensive assessment including physical and mental health |  | U | Diagnosis/ Assessment | 147 | 147 | 87.1% (79.1, 92.9) |
|  |  | COGI24 | Residents with behaviours and psychological symptoms of dementia (BPSD) had a comprehensive assessment including level of pain or discomfort |  | U | Diagnosis/ Assessment | 146 | 146 | 93.8% (88.6, 97.1) |
|  |  | COGI25 | Residents with behaviours and psychological symptoms of dementia (BPSD) had a comprehensive assessment including side effects of medication |  | U | Diagnosis/ Assessment | 145 | 145 | 14.3% (6.5, 26.0) |
|  |  | COGI26 | Residents with behaviours and psychological symptoms of dementia (BPSD) had a comprehensive assessment including the influence of religious and spiritual beliefs and cultural norms |  | U | Diagnosis/ Assessment | 146 | 146 | 82.9% (66.8, 93.3) |
|  |  | COGI27 | Residents with behaviours and psychological symptoms of dementia (BPSD) had a comprehensive assessment including physical, environmental and interpersonal factors |  | U | Diagnosis/ Assessment | 146 | 146 | 84.7% (72.8, 92.9) |
|  |  | COGI28 | Residents with behaviours and psychological symptoms of dementia (BPSD) had a comprehensive assessment including carer(s) health and communication style when interacting with have the resident. |  | U | Diagnosis/ Assessment | 146 | 146 | 98.6% (95.2, 99.8) |
| 19 | Residents who have behaviours and psychological symptoms of dementia (BPSD) should have a care plan that incorporates a range of non-pharmacological approaches, selected according to the assessment of the BPSD | COGI29 | Residents with behaviours and psychological symptoms of dementia (BPSD) had a care plan that incorporated a range of non-pharmacological approaches, selected according to the assessment of the BPSD | (34, 61, 76, 78, 83-85) | U | Treatment | 146 | 146 | 98.1% (94.3, 99.7) |
| 20 | Residents who have behaviours and psychological symptoms of dementia (BPSD) should have a care plan developed in consultation with the resident and their family. | COGI30 | Residents with behaviours and psychological symptoms of dementia (BPSD) who had a care plan had it developed in consultation with the resident and their family / substitute decision maker. | (76, 78) | U | Treatment; Resident/ Family engagement | 141 | 141 | 63.7% (47.9, 77.6) |
| 21 | Residents^1^ who have behaviours and psychological symptoms of dementia (BPSD) who are being deprescribed antipsychotics should be provided with information about deprescribing. | COGI31 | Residents^1^ with behaviours and psychological symptoms of dementia (BPSD) who are being deprescribed antipsychotics were provided with information about deprescribing | (86) | U | Information provision; Resident/ Family engagement | 8 | 8 | nc |
| 22 | Residents who have moderate to severe behaviours and psychological symptoms of dementia (BPSD) which puts themselves or others at risk, should be referred to a specialist service within 24 hours. | COGI32 | Residents who have moderate to severe behaviours and psychological symptoms of dementia (BPSD) which puts themselves or others at risk, were referred to a specialist service within 24 hours | (76) | U | Referral/ Consultation | 12 | 14 | nc |
| **Depression** | | | | | | | | | |
| 1 | Residents who have suspected depression should receive a comprehensive assessment. | DEPR01 | Residents who have suspected depression received a comprehensive assessment | (62, 65, 66, 87) | U | Diagnosis/ Assessment | 9 | 9 | nc |
| 2 | Residents newly diagnosed with depression should be assessed for risk of suicide. | DEPR02 | Residents who are newly diagnosed with depression were assessed for risk of suicide | (34, 65, 66) | U | Diagnosis/ Assessment | 4 | 4 | nc |
| 3 | Residents who have depression and present considerable immediate risk to themselves or others should be referred urgently (within 24 hours) to specialist mental health services | DEPR03 | Residents who have depression and present considerable immediate risk to themselves or others were referred urgently (within 24 hours) to specialist mental health services | (62, 66) | U | Referral/ Consultation | 5 | 5 | nc |
| 4 | Residents who have depression should have a comprehensive multidisciplinary care plan. | DEPR04 | Residents who have depression had a comprehensive multidisciplinary care plan | (34, 65, 66) | U | Treatment | 145 | 145 | 17.2% (9.0, 28.5) |
| 5 | Residents^1^ who are newly diagnosed with depression should receive information on depression and the treatment plan. | DEPR05 | Residents^1^ who are newly diagnosed with depression received information on depression and the treatment plan | (62) | U | Information provision; Resident/ Family engagement | 4 | 4 | nc |
| 6 | Residents who are newly prescribed antidepressants should be monitored for side effects weekly for the first four weeks. | DEPR06 | Residents who have depression and were newly prescribed antidepressants were monitored for side effects weekly for the first four weeks | (88) | U | Monitoring/ Review | 6 | 6 | nc |
| 7 | Residents prescribed antidepressants should be monitored for side effects monthly. | DEPR07 | Residents who have depression and have been receiving antidepressants for four weeks were monitored for side effects monthly | (88) | U | Monitoring/ Review | 105 | 105 | 0.6% (0.0, 4.7) |
| 8 | Residents who are newly diagnosed with depression and are prescribed medication should receive one of the following as a first line treatment:  - Mirtazapine OR - Citalopram/escitalopram OR - Desvenlafaxine OR - Duloxetine OR - Sertraline OR - Venlafaxine OR - Fluoxetine OR - Fluvoxamine OR - Paroxetine OR - Moclobemide | DEPR08 | Residents who are newly diagnosed with depression and are prescribed/ medication received one of the following as a first line treatment:   - Mirtazapine OR  - Citalopram/ escitalopram OR  - Desvenlafaxine OR  - Duloxetine OR  - Sertraline OR  - Venlafaxine OR  - Fluoxetine OR  - Fluvoxamine OR  - Paroxetine OR  - Moclobemide | (87, 88) | U | Treatment | 2 | 2 | nc |
| 9 | Residents who have severe depression should be referred to psychiatric services within 24 hours. | DEPR09 | Residents who have severe depression were referred to psychiatric services within 24 hours. | (62-64) | U | Referral/ Consultation | 4 | 4 | nc |
| 10 | Residents who have severe depression should have treatment started immediately. | DEPR10 | Residents who have severe depression had treatment started immediately. | (89) | U | Treatment | 5 | 5 | nc |
| 11 | Residents who have moderate or severe depression should be provided with a combination of antidepressant medication and a psychological intervention (cognitive behavioural therapy or interpersonal psychotherapy). | DEPR11 | Residents who have moderate or severe depression were provided with a combination of antidepressant medication and a psychological intervention (cognitive behavioural therapy or interpersonal psychotherapy). | (66) | U | Treatment | 56 | 56 | 10.6% (3.3, 23.5) |
| **Dysphagia** | | | | | | | | | |
| 1 | Residents who have dysphagia, and a significant change in clinical condition, should receive an assessment by a speech pathologist within a week. | DYSP01 | Residents who have dysphagia, and a significant change in clinical condition, received an assessment by a speech pathologist within a week. | (28, 50, 55) | U | Diagnosis/ Assessment | 29 | 30 | 38.0% (7.8, 77.5) |
| 2 | Residents who have dysphagia should have a dietitian assess nutrition and hydration requirements within a week. | DYSP02 | Residents who have dysphagia had a dietitian assess nutrition and hydration requirements within a week. | (50) | U | Diagnosis/ Assessment | 29 | 29 | 19.1% (1.9, 56.8) |
| 3 | Residents who have dysphagia should receive a dysphagia care plan. | DYSP03 | Residents with dysphagia had a dysphagia care plan. | (50, 55) | U | Treatment | 94 | 94 | 88.2% (74.0, 96.2) |
| 4 | Residents who have a choking incident should receive or have a review of a choking/dysphagia care plan | DYSP04 | Residents who have a choking incident received or had a review of a choking/dysphagia care plan | (55) | U | Treatment | 6 | 6 | nc |
| 5 | Residents who have acute dysphagia should receive immediate evaluation and intervention (within 6 hours.) | DYSP05 | Residents with acute dysphagia had immediate evaluation and intervention (within 6 hours.) | (28) | U | Treatment | 11 | 12 | nc |
| 6 | Residents who have new onset signs of dysphagia, choking risk OR who have a choking incident should receive a referral to: - a GP OR - a speech pathologist | DYSP06 | Residents who have new onset signs of dysphagia, choking risk OR who have a choking incident received a referral to a GP OR a speech pathologist | (55) | U | Referral/ Consultation | 27 | 27 | 84.0% (56.8, 97.3) |
| 7 | Residents who have a choking incident should be monitored for 3 days for: - swallowing difficulties AND - adequacy of food and fluid intake AND - signs of chest infection. | DYSP07 | Residents who had a choking incident were monitored for 3 days for swallowing difficulties | (55) | U | Monitoring/ Review | 7 | 7 | nc |
|  |  | DYSP08 | Residents who had a choking incident were monitored for 3 days for adequacy of food and fluid intake. |  | U | Monitoring/ Review | 7 | 7 | nc |
|  |  | DYSP09 | Residents who had a choking incident were monitored for 3 days for signs of chest infection. |  | U | Monitoring/ Review | 6 | 6 | nc |
| **End of life care** | | | | | | | | | |
| 1 | All Residents should have a holistic assessment of their End of life and palliative care needs reviewed at regular intervals. | EOLC01 | Residents had a holistic assessment of their end-of-life needs reviewed at regular intervals | (35, 36, 38) | U | Monitoring/ Review | 278 | 278 | 90.0% (82.9, 94.9) |
| 2 | Residents should have a clinical care plan relating to end of life. | EOLC02 | Residents received a clinical care plan relating to end of life. | (34) | U | Treatment | 273 | 273 | 25.2% (15.7, 36.8) |
| 3 | Residents should be supported to develop their advance care plan. | EOLC03 | Residents were supported to develop their advance care plan. | (34-36, 90-92) | U | Treatment; Resident/ Family engagement | 262 | 262 | 13.0% (6.4, 22.7) |
| 4 | Residents should be involved in making end-of-life decisions and developing care plans in partnership with: - their families and - the interdisciplinary team. | EOLC04 | Residents were involved in making end-of-life decisions and developing care plans in partnership with: - their families and - the interdisciplinary team. | (36, 90-93) | U | Treatment; Resident/ Family engagement | 266 | 266 | 18.9% (11.6, 28.1) |
| 5 | Residents who have advanced dementia should be assessed using clinical indicators of mortality to identify approach to end of life. | EOLC05 | Residents who have advanced dementia were assessed using clinical indicators of mortality to identify approach to end of life. | (94) | U | Diagnosis/ Assessment | 68 | 68 | 10.2% (0.4, 41.6) |
| 6 | Residents who have established terminal distress/agitation should have midazolam or clonazepam as first line treatment. | EOLC06 | Residents who have established terminal distress/agitation received midazolam or clonazepam as first line treatment. | (95) | U | Treatment | 35 | 36 | 99.0% (88.4, 100.0) |
| 7 | Residents^1^ who are receiving end of life care should be provided with information including: - their clinical condition and its severity or stage AND - the expected disease trajectory AND - the available treatments AND  - the likelihood of response to such treatments. | EOLC07 | Residents^1^ who are receiving end of life care were provided with information including their clinical condition and its severity or stage | (34-36, 90-93, 96) | U | Information provision; Resident/ Family engagement | 55 | 55 | 72.1% (54.1, 86.1) |
|  |  | EOLC08 | Residents^1^ who are receiving end of life care were provided with information including the expected disease trajectory |  | U | Information provision; Resident/ Family engagement | 55 | 55 | 51.7% (34.3, 68.8) |
|  |  | EOLC09 | Residents^1^ who are receiving end of life care were provided with information including the available treatments |  | U | Information provision; Resident/ Family engagement | 54 | 55 | 66.3% (48.4, 81.3) |
|  |  | EOLC10 | Residents^1^ who are receiving end of life care were provided with information including the likelihood of response to such treatments. |  | U | Information provision ; Resident/ Family engagement | 53 | 53 | 38.3% (22.6, 56.0) |
| 8 | Residents who are dying, should not have enteral or parental feeding. | EOLC11 | Residents who are dying had enteral or parental feeding. | (50) | O | Treatment | 53 | 53 | 100.0% (93.3, 100.0) |
| 9 | Residents^1^ who are dying should be provided with: - accurate information about their prognosis (unless they do not wish to be informed), explaining any uncertainty and how this will be managed AND - an opportunity to talk about any fears and anxieties, and to ask questions about their care in the last days of life AND - information about how to contact members of their care team AND  - opportunities for further discussion with a member of their care team. | EOLC12 | Residents^1^ who are dying were provided with accurate information about their prognosis (unless they do not wish to be informed), explaining any uncertainty and how this will be managed | (34, 35, 37, 91-93, 95) | U | Information provision; Resident/ Family engagement | 56 | 56 | 46.7% (30.1, 63.8) |
|  |  | EOLC13 | Residents^1^ who are dying were provided with an opportunity to talk about any fears and anxieties, and to ask questions about their care in the last days of life |  | U | Resident/Family engagement | 57 | 57 | 53.8% (35.9, 71.0) |
|  |  | EOLC14 | Residents^1^ who are dying were provided with information about how to contact members of their care team |  | U | Information provision; Resident/ Family engagement | 57 | 57 | 23.2% (10.3, 41.4) |
|  |  | EOLC15 | Residents^1^ who are dying were provided with opportunities for further discussion with a member of their care team. |  | U | Resident/Family engagement | 57 | 57 | 43.0% (26.1, 61.2) |
| 10 | Residents who are dying should have an individualised care plan including: - personal goals and wishes AND - preferred care setting AND - current and anticipated care needs (including preferences for symptom management, needs for care after death) AND - resource needs. | EOLC16 | Residents who are dying had an individualised care plan including personal goals and wishes | (34, 35, 37, 90-93, 95) | U | Treatment; Resident/ Family engagement | 57 | 57 | 83.0% (68.4, 92.7) |
|  |  | EOLC17 | Residents who are dying had an individualised care plan including preferred care setting |  | U | Treatment | 58 | 58 | 81.9% (69.1, 91.0) |
|  |  | EOLC18 | Residents who are dying had an individualised care plan including current and anticipated care needs (including preferences for symptom management, needs for care after death) |  | U | Treatment | 56 | 56 | 75.0% (56.7, 88.5) |
|  |  | EOLC19 | Residents who are dying had an individualised care plan including resource needs. |  | U | Treatment | 53 | 53 | 44.5% (30.5, 59.2) |
| 11 | Residents who are dying should be prescribed anticipatory medicines with indications for use, and a range of doses and routes of administration. | EOLC20 | Residents who are dying were prescribed anticipatory medicines with documentation of indications for use, and a range of doses and routes of administration. | (36, 37, 91, 93, 95-97) | U | Treatment | 53 | 53 | 90.5% (79.2, 96.8) |
| 12 | Residents who are dying and are receiving anticipatory medicines should be monitored two hourly | EOLC21 | Residents who are dying and are receiving anticipatory medicines were monitored two hourly | (91, 93, 97) | U | Monitoring/ Review | 50 | 50 | 55.5% (38.4, 71.7) |
| 13 | Residents who are dying should have their symptoms and care plan reviewed before anticipatory medicines are administered. | EOLC22 | Residents who are dying had their symptoms and care plan reviewed before anticipatory medicines were administered. | (91, 97) | U | Treatment | 54 | 54 | 47.9% (30.8, 65.5) |
| 14 | Residents who are dying should have their symptoms reassessed daily. | EOLC23 | Residents who are dying had their symptoms reassessed daily. | (34, 91, 97) | U | Monitoring/ Review | 55 | 57 | 80.9% (65.5, 91.6) |
| 15 | Residents who are dying should be supported to receive oral food and fluids. | EOLC24 | Residents who are dying were supported to receive oral food and fluids. | (90, 91, 95) | U | Treatment | 52 | 52 | 84.7% (68.7, 94.5) |
| 16 | Residents who are dying and have nausea and vomiting should be treated with: - a long-acting anti-emetic subcut or  - a suitable anti-emetic as a 24-hour CSCI | EOLC25 | Residents who are dying and have nausea and vomiting were treated with: - a long-acting anti-emetic subcut or  - a suitable anti-emetic as a 24-hour CSCI | (93, 95-97) | U | Treatment | 18 | 20 | nc |
| 17 | Residents who are dying and have persistent breathlessness or respiratory distress should be treated with: - Morphine OR  - Hydromorphone | EOLC26 | Residents who are dying and have persistent breathlessness or respiratory distress were treated with: - Morphine OR - Hydromorphone | (95, 97) | U | Treatment | 25 | 25 | 80.4% (50.9, 96.2) |
| 18 | Residents who are dying and have pain should be treated with:  - morphine OR - hydromorphone | EOLC27 | Residents who are dying and have pain were treated with:  - morphine OR - hydromorphone | (96, 97) | U | Treatment | 53 | 55 | 94.1% (82.4, 99.0) |
| 19 | Residents who are dying and have restlessness and agitation should be treated with a benzodiazepine. | EOLC28 | Residents who are dying and have restlessness and agitation were treated with a benzodiazepine. | (97) | U | Treatment | 32 | 34 | 94.2% (80.5, 99.3) |
| 20 | Residents who are dying and have had nutritional support stopped, should have nasogastric tubes removed. | EOLC29 | Residents who are dying and have had nutritional support stopped had nasogastric tubes removed. | (92) | U | Treatment | 2 | 2 | nc |
| 21 | Residents who are dying and have uncontrolled symptoms (i.e., pain or nausea/vomiting) should have urgent palliative care service/specialist advise sought within 24 hours. | EOLC30 | Residents who are dying and have uncontrolled symptoms (i.e., pain or nausea/vomiting) had urgent palliative care service/specialist advise sought within 24 hours. | (93, 97) | U | Referral/ Consultation | 3 | 3 | nc |
| 22 | Residents who are dying should be provided with comfort care measures. | EOLC31 | Residents who are dying were provided with comfort care measures. | (93) | U | Treatment | 55 | 55 | 97.1% (88.6, 99.8) |
| 23 | Residents who are in the last days of life should have an assessment addressing physical, emotional, psychological and spiritual needs. | EOLC32 | Residents who are in the last days of life had an assessment addressing physical, emotional, psychological and spiritual needs. | (36, 90-93) |  | Diagnosis/ Assessment | 55 | 55 | 38.3% (22.2, 56.6) |
| **Hearing and vision** | | | | | | | | | |
| 1 | Residents who present for the first time with hearing difficulties should: - have an otoscopic examination to exclude impacted wax and acute infection  - be referred for audiological assessment | HEVI01 | Residents presenting for the first time with hearing difficulties had an otoscopic examination to exclude impacted wax and acute infection. | (98-100) | U | Diagnosis/ Assessment | NA | NA | NA |
|  |  | HEVI02 | Residents presenting for the first time with hearing difficulties were referred for audiological assessment |  | U | Referral/ Consultation | NA | NA | NA |
| 2 | Residents presenting with new hearing loss should have a history of the hearing loss taken including:  - duration AND - unilateral/bilateral AND - any associated otological or neurological symptoms or signs | HEVI03 | Residents presenting with new hearing loss had a history of the\ hearing loss taken including:   - duration AND  - unilateral/bilateral\ AND  - any associated otological or neurological symptoms or signs | (99) | U | Diagnosis/ Assessment | NA | NA | NA |
| 3 | Residents who have hearing loss should be provided with information about its management. | HEVI04 | Residents who have hearing loss were provided with information about its management. | (98) | U | Information provision; Resident/ Family engagement | NA | NA | NA |
| 4 | Residents who have hearing loss that affects their ability to communicate should be referred for assessment for hearing aids or other hearing devices within one month. | HEVI05 | Residents who have hearing loss that affects their ability to communicate were referred for assessment for hearing aids or other hearing devices within one month. | (98) | U | Referral/ Consultation | NA | NA | NA |
| 5 | Residents who have any new vision loss or sudden change in vision should be referred for an assessment by an eye care specialist within one week. | HEVI06 | Residents who have any new vision loss or sudden change in vision were referred for an assessment by an eye care specialist within one week. | (101) | U | Referral/ Consultation | NA | NA | NA |
| **Infection** | | | | | | | | | |
| 1 | Residents should receive (unless medically contraindicated) current vaccinations for: - tetanus AND - diphtheria AND - influenza AND - pertussis AND - pneumococcus. | INFC01 | Residents had (unless medically contraindicated) current vaccinations for tetanus | (27, 34, 102) | U | Treatment | 224 | 224 | 4.6% (1.6, 9.9) |
|  |  | INFC02 | Residents had (unless medically contraindicated) current vaccinations for diphtheria |  | U | Treatment | 224 | 224 | 4.2% (1.4, 9.7) |
|  |  | INFC03 | Residents had (unless medically contraindicated) current vaccinations for influenza |  | U | Treatment | 202 | 202 | 85.9% (75.5, 93.0) |
|  |  | INFC04 | Residents had (unless medically contraindicated) current vaccinations for pertussis |  | U | Treatment | 225 | 225 | 3.5% (0.8, 9.1) |
|  |  | INFC05 | Residents had (unless medically contraindicated) current vaccinations for pneumococcus |  | U | Treatment | 224 | 224 | 25.1% (14.7, 38.2) |
| 2 | Residents who have a suspected infection should have an initial clinical evaluation documented including: - HR AND - BP AND - temperature AND - pulse oximetry AND - respiratory rate AND - delirium screen AND - cognition appropriate pain assessment AND  - conscious level (Alert, Verbal, Pain, Unresponsive (AVPU))  - BGL. | INFC06 | Residents who have a suspected infection had an initial clinical evaluation documented including HR | (103) | U | Diagnosis/ Assessment | 87 | 119 | 53.3% (37.0, 69.1) |
|  |  | INFC07 | Residents who have a suspected infection had an initial clinical evaluation documented including BP |  | U | Diagnosis/ Assessment | 87 | 119 | 55.4% (39.0, 71.1) |
|  |  | INFC08 | Residents who have a suspected infection had an initial clinical evaluation documented including temperature |  | U | Diagnosis/ Assessment | 87 | 119 | 71.3% (56.0, 83.7) |
|  |  | INFC09 | Residents who have a suspected infection had an initial clinical evaluation documented including pulse oximetry |  | U | Diagnosis/ Assessment | 87 | 119 | 46.6% (30.8, 62.9) |
|  |  | INFC10 | Residents who have a suspected infection had an initial clinical evaluation documented including respiratory rate |  | U | Diagnosis/ Assessment | 87 | 119 | 53.5% (37.2, 69.3) |
|  |  | INFC11 | Residents who have a suspected infection had an initial clinical evaluation documented including a delirium screen |  | U | Diagnosis/ Assessment | 87 | 119 | 0.6% (0.0, 4.1) |
|  |  | INFC12 | Residents who have a suspected infection had an initial clinical evaluation documented including a cognition appropriate pain assessment |  | U | Diagnosis/ Assessment | 87 | 119 | 21.5% (11.5, 34.8) |
|  |  | INFC13 | Residents who have a suspected infection had an initial clinical evaluation documented including conscious level (Alert, Verbal, Pain, Unresponsive (AVPU)) |  | U | Diagnosis/ Assessment | 87 | 119 | 7.4% (2.2, 17.0) |
|  |  | INFC14 | Residents who have a suspected infection had an initial clinical evaluation documented including BGL. |  | U | Diagnosis/ Assessment | 86 | 118 | 9.8% (3.8, 20.0) |
| 3 | Residents suspected of, or who have symptoms of, urinary tract infection should have a full clinical assessment including vital signs prior to diagnosis. | INFC15 | Residents suspected of, or who have symptoms of, urinary tract infection had a full clinical assessment including vital signs prior to diagnosis. | (104, 105) | U | Diagnosis/ Assessment | 61 | 76 | 23.5% (10.7, 41.2) |
| 4 | Residents who have symptoms of a urinary tract infection should have a urine sample taken (to test for signs of infection or other abnormality) within 24 hours. | INFC16 | Residents who have symptoms of a urinary tract infection had a urine sample taken (to test for signs of infection or other abnormality) within 24 hours. | (34, 70, 105) | U | Diagnosis/ Assessment | 59 | 73 | 92.2% (83.5, 97.2) |
| 5 | Female residents who have symptoms of voiding dysfunction or recurrent urinary tract infection should have their post-void residual volume measured by bladder scan or catheterisation. | INFC17 | Female residents who have symptoms of voiding dysfunction or recurrent urinary tract infection had their post-void residual volume measured by bladder scan or catheterisation. | (70) | U | Diagnosis/ Assessment | 47 | 48 | 0.0% (0.0, 7.4) |
| 6 | Residents who have asymptomatic bacteriuria or asymptomatic pyuria should not receive treatment with an antibiotic. | INFC18 | Residents who have asymptomatic bacteriuria or asymptomatic pyuria received antibiotic treatment. | (102, 105) | O | Treatment | 4 | 4 | nc |
| 7 | Residents newly diagnosed with lower urinary tract infection should receive antibiotics (at time of diagnosis) | INFC19 | Residents newly diagnosed with lower urinary tract infection received antibiotics (at time of diagnosis) | (34, 102, 105) | U | Treatment | 41 | 48 | 99.9% (92.4, 100.0) |
| 8 | Residents who have erysipelas or cellulitis should not have:  - routine blood culture OR - aspiration culture OR - punch biopsy culture | INFC20 | Residents who have erysipelas or cellulitis had routine blood culture | (106) | O | Diagnosis/ Assessment | 12 | 12 | nc |
|  |  | INFC21 | Residents who have erysipelas or cellulitis had aspiration culture |  | O | Diagnosis/ Assessment | 12 | 12 | nc |
|  |  | INFC22 | Residents who have erysipelas or cellulitis had punch biopsy culture |  | O | Diagnosis/ Assessment | 12 | 12 | nc |
| 9 | Residents who have erysipelas should receive penicillin or cefalexin, clindamycin, cefazolin. | INFC23 | Residents who have erysipelas had penicillin or cefalexin, clindamycin, cefazolin. | (106, 107) | U | Treatment | 4 | 5 | nc |
| 10 | Residents who have cellulitis should receive: - dicloxacillin OR - trimethoprim sulfamethoxazole OR - flucloxacillin OR - clindamycin | INFC24 | Residents who have cellulitis received: - dicloxacillin OR - trimethoprim sulfamethoxazole OR - flucloxacillin OR - clindamycin | (106, 107) | U | Treatment | 9 | 9 | nc |
| 11 | Residents who have erysipelas or cellulitis without complications should receive antibiotics for 5 days. | INFC25 | Residents who have erysipelas or cellulitis without complications received antibiotics for 5 days. | (106) | U | Treatment | 12 | 13 | nc |
| 12 | Residents who have suspected pneumonia should have: - pulse oximetry AND - chest radiograph AND - full blood count with differential AND - blood urea. | INFC26 | Residents who have suspected pneumonia had pulse oximetry | (27, 103) | U | Diagnosis/ Assessment | 12 | 12 | nc |
|  |  | INFC27 | Residents who have suspected pneumonia had chest radiograph |  | U | Diagnosis/ Assessment | 10 | 10 | nc |
|  |  | INFC28 | Residents who have suspected pneumonia had full blood count with differential |  | U | Diagnosis/ Assessment | 10 | 10 | nc |
|  |  | INFC29 | Residents who have suspected pneumonia had blood urea. |  | U | Diagnosis/ Assessment | 10 | 10 | nc |
| 13 | Residents who have pneumonia (which is treated in the RACF) should receive antibiotic treatment with Amoxicillin OR if penicillin allergies are present doxycycline or cefuroxime | INFC30 | Residents who have pneumonia (which is treated in the RACF) received antibiotic treatment with Amoxicillin; OR, if penicillin allergies are present, doxycycline or cefuroxime | (102) | U | Treatment | 10 | 10 | nc |
| 14 | Residents who have severe aspiration pneumonia are transferred to hospital. | INFC31 | Residents who have severe aspiration pneumonia were transferred to hospital. | (102) | U | Treatment | 2 | 2 | nc |
| 15 | Residents receiving treatment for pneumonia should be assessed daily. | INFC32 | Residents receiving treatment for pneumonia were assessed daily. | (102) | U | Monitoring/ Review | 11 | 11 | nc |
| 16 | Residents who have suspected influenza should have a nose and/or throat swab for laboratory testing. | INFC33 | Residents who have suspected influenza had a nose and/or throat swab for laboratory testing. | (27) | U | Diagnosis/ Assessment | 0 | 0 | nc |
| **Medication** | | | | | | | | | |
| 1 | Residents should have a medication review when they: - have worsening health OR - have signs of administration problems OR - are on multiple psychotropic drugs OR - when a new medicine is ordered. | MEDI01 | Residents who have worsening health had a medication review | (33, 34, 71) | U | Monitoring/ Review | 90 | 111 | 26.4% (14.8, 41.0) |
|  |  | MEDI02 | Residents who have signs of administration problems had a medication review |  | U | Monitoring/ Review | 57 | 58 | 72.1% (51.0, 87.9) |
|  |  | MEDI03 | Residents on multiple psychotropic drugs had a medication review |  | U | Monitoring/ Review | 101 | 104 | 58.9% (38.9, 76.9) |
|  |  | MEDI04 | Residents who had a new medicine ordered had a medication review |  | U | Monitoring/ Review | 141 | 167 | 19.5% (10.1, 32.1) |
| 2 | Residents^1^ should be involved in their medication review. | MEDI05 | Residents^1^ who had a medication review were involved in their medication review | (71, 108, 109) | U | Monitoring/ Review; Resident/ Family engagement | 90 | 94 | 40.7% (22.9, 60.5) |
| 3 | Residents^1^ who are newly prescribed a medication should be provided with information and education about ~~t~~heir medications including: - consumer medicine information - current treatment and any changes made - possible drug-related problems that might occur  - what to do in case side-effects occur or a dose is forgotten - checking that they understand the treatment and how to implement it. | MEDI06 | Residents^1^ who are newly prescribed a medication were provided with information and education about their medications including: - consumer medicine information - current treatment and any changes made - possible drug-related problems that might occur  - what to do in case side-effects occur or a dose is forgotten - checking that they understand the treatment and how to implement it. | (33, 71, 110, 111) | U | Information provision; Resident/ Family engagement | 138 | 150 | 9.2% (2.2, 23.1) |
| 4 | Residents who are newly prescribed a medication should receive a monitoring plan. | MEDI07 | Residents who are newly prescribed a medication received a monitoring plan. | (33, 34, 71, 110-112) | U | Monitoring/ Review | 142 | 154 | 21.9% (10.6, 37.4) |
| 5 | Residents prescribed benzodiazepines OR antipsychotics should have a written tapering plan | MEDI08 | Residents prescribed benzodiazepines OR antipsychotics had a written tapering plan | (34) | U | Treatment | 78 | 79 | 11.8% (1.3, 37.5) |
| 6 | Residents with diabetes, who are on insulin therapy or glucose lowering medications should receive a hypoglycaemia management plan. | MEDI09 | Residents with diabetes, who are on insulin therapy or glucose lowering medications received a hypoglycaemia management plan. | (113) | U | Treatment | 32 | 32 | 66.1% (40.2, 86.4) |
| 7 | Residents who are on psychotropic medications and have fallen should have these reviewed within a week of fall. | MEDI10 | Residents who are on psychotropic medications and have fallen had these reviewed within a week of fall. | (71) | U | Monitoring/ Review | 70 | 92 | 17.7% (4.6, 40.7) |
| **Mobility and Falls** | | | | | | | | | |
| 1 | Residents should be encouraged to attend to their activities of daily living. | MOBI01 | Residents were encouraged to attend to their activities of daily living. | (41) | U | Treatment | 267 | 267 | 86.1% (80.4, 90.6) |
| 2 | Residents should be prescribed strength and balance training for falls prevention in their current care plan. | MOBI02 | Residents were prescribed strength and balance training for falls prevention in their current care plan. | (29-31, 42, 114) | U | Treatment | 234 | 234 | 59.9% (48.8, 70.3) |
| 3 | Residents prescribed an exercise program should have it reviewed 3 monthly. | MOBI03 | Residents prescribed an exercise program had it reviewed 3 monthly. | (29) | U | Monitoring/ Review | 82 | 82 | 17.7% (6.3, 35.8) |
| 4 | Residents should receive vitamin D supplementation. | MOBI04 | Residents received vitamin D supplementation. | (29, 32, 41, 114) | U | Treatment | 266 | 266 | 68.0% (56.7, 78.0) |
| 5 | Residents at medium/high risk of falling should receive a multifactorial intervention. | MOBI05 | Residents at medium/high risk of falling received a multifactorial intervention. | (29-31, 34, 41, 42, 114, 115) | U | Treatment | 240 | 240 | 95.0% (85.9, 99.0) |
| 6 | Residents unable to mobilise on their own should be prescribed walking or mobility aids and/or assistance. | MOBI06 | Residents unable to mobilise on their own were prescribed walking or mobility aids and/or assistance. | (41) | U | Treatment | 211 | 211 | 100.0% (98.3, 100.0) |
| 7 | Residents who have vision deficits should be encouraged to wear their prescription glasses. | MOBI07 | Residents who have vision deficits were encouraged to wear their prescription glasses. | (29) | U | Treatment | 95 | 95 | 79.5% (64.9, 90.0) |
| 8 | Residents who have corrected visual acuity worse than 6/24 should have an environmental assessment and modification undertaken. | MOBI08 | Residents who have corrected visual acuity worse than 6/24 had an environmental assessment and modification undertaken. | (29) | U | Diagnosis/ Assessment; Treatment | 33 | 33 | 27.2% (8.1, 55.7) |
| 9 | Residents who have recurrent falls, multiple risk factors or complex needs should be referred to the appropriate clinician(s). | MOBI09 | Residents who have recurrent falls, multiple risk factors or complex needs were referred to the appropriate clinician(s). | (30, 31) | U | Referral/ Consultation | 118 | 140 | 89.3% (81.6, 94.6) |
| 10 | Residents post-fall should have details of the fall taken | MOBI10 | Residents post-fall had details of the fall taken | (29-31, 34, 41, 42) | U | Diagnosis/ Assessment | 110 | 220 | 98.6% (94.9, 99.8) |
| 11 | Residents post-fall should receive a comprehensive physical assessment (within a week of the fall) of their: - gait AND - balance AND - transfers and mobility AND - lower limb joint function AND - lower limb muscle strength AND - feet (toe/nail deformities, ulcers, corn, calluses, bunion). | MOBI11 | Residents post-fall received a comprehensive physical assessment (within a week of the fall) of their gait | (29-32, 34, 41, 42, 116) | U | Diagnosis/ Assessment | 97 | 192 | 35.2% (22.6, 49.6) |
|  |  | MOBI12 | Residents post-fall received a comprehensive physical assessment (within a week of the fall) of their balance |  | U | Diagnosis/ Assessment | 97 | 193 | 33.0% (20.5, 47.5) |
|  |  | MOBI13 | Residents post-fall received a comprehensive physical assessment (within a week of the fall) of their transfers and mobility |  | U | Diagnosis/ Assessment | 104 | 211 | 49.7% (36.7, 62.6) |
|  |  | MOBI14 | Residents post-fall received a comprehensive physical assessment (within a week of the fall) of their lower limb joint function |  | U | Diagnosis/ Assessment | 97 | 193 | 19.7% (9.1, 34.8) |
|  |  | MOBI15 | Residents post-fall received a comprehensive physical assessment (within a week of the fall) of their lower limb muscle strength |  | U | Diagnosis/ Assessment | 97 | 193 | 22.3% (11.1, 37.4) |
|  |  | MOBI16 | Residents post-fall received a comprehensive physical assessment (within a week of the fall) of their feet (toe/nail deformities, ulcers, corn, calluses, bunion). |  | U | Diagnosis/ Assessment | 98 | 193 | 8.8% (2.0, 23.0) |
| 12 | Residents post-fall should receive a functional assessment (within a week of the fall). | MOBI17 | Residents post-fall received a functional assessment (within a week of the fall). | (29, 31, 32, 41, 42) | U | Diagnosis/ Assessment | 106 | 214 | 26.7% (17.2, 38.2) |
| 13 | Residents post-fall should receive a medication review (within a week of the fall). | MOBI18 | Residents post-fall received a medication review (within a week of the fall). | (29-32, 34, 41, 42) | U | Monitoring/ Review | 104 | 212 | 13.7% (5.6, 26.4) |
| 14 | Residents post-fall should receive an assessment of their psychosocial status (within a week of the fall). | MOBI19 | Residents post-fall received an assessment of their psychosocial status (within a week of the fall). | (29, 31, 32, 41, 42) | U | Diagnosis/ Assessment | 106 | 214 | 14.9% (4.4, 33.2) |
| 15 | Residents post-fall who have sustained a head injury or may have hit their head during an unobserved fall, should have neurological observations (Glasgow coma scale - GCS) hourly for four hours. | MOBI20 | Residents post-fall who have sustained a head injury or may have hit their head during an unobserved fall, had neurological observations (Glasgow coma scale - GCS) hourly for four hours. | (29) | U | Monitoring/ Review | 95 | 187 | 73.2% (60.9, 83.3) |
| **Nutrition and Hydration** | | | | | | | | | |
| 1 | Residents should receive monthly screening for malnutrition using a validated tool. | NUHY01 | Residents received monthly screening for malnutrition using a validated tool. | (50, 53) | U | Diagnosis/ Assessment | 270 | 270 | 12.0% (6.4, 19.9) |
| 2 | Residents should be weighed monthly. | NUHY02 | Residents were weighed monthly. | (26, 50) | U | Diagnosis/ Assessment | 271 | 405 | 99.3% (98.0, 99.9) |
| 3 | Residents should have a current nutrition care plan. | NUHY03 | Residents had a current nutrition care plan. | (50) | U | Treatment | 272 | 272 | 99.3% (97.4, 99.9) |
| 4 | Residents should receive quarterly review of their nutrition care plan. | NUHY04 | Residents received quarterly review of their nutrition care plan. | (50) | U | Treatment; Monitoring/ Review | 267 | 267 | 22.9% (14.9, 32.8) |
| 5 | Residents should have interventions implemented if:  - any unplanned and unexpected weight loss is detected OR  - the BMI is less than 18.5 OR - they leave one or more meal per day - there is sudden weight loss. | NUHY05 | Residents who had any unplanned and unexpected weight loss OR BMI less than 18.5 OR leave one or more meal per day OR have sudden weight loss had interventions implemented. | (26) | U | Treatment | 76 | 76 | 81.5% (58.5, 94.9) |
| 6 | Residents who have unplanned weight loss or being at risk of weight loss, should be weighed more than once a month. | NUHY06 | Residents who have unplanned weight loss or being at risk of weight loss, were weighed more than once a month. | (26) | U | Monitoring/ Review | 108 | 108 | 20.7% (9.8, 35.9) |
| 7 | Residents^1^ who have unplanned weight loss should receive written information on nutrition and maintaining nutritional status. | NUHY07 | Residents^1^ who have unplanned weight loss received written information on nutrition and maintaining nutritional status. | (26) | U | Information provision; Resident/ Family engagement | 69 | 69 | 1.4% (0.0, 7.7) |
| 8 | Residents who have unplanned weight loss or are at risk of unplanned weight loss should receive nutritional supplementation. | NUHY08 | Residents who have unplanned weight loss or are at risk of unplanned weight loss received nutritional supplementation. | (26) | U | Treatment | 118 | 118 | 87.5% (76.1, 94.8) |
| 9 | Residents who have unplanned weight loss or are at risk of weight loss, should receive referral to: - a GP AND - a dietitian | NUHY09 | Residents who have unplanned weight loss or are at risk of weight loss, received referral to a GP | (26) | U | Referral/ Consultation | 111 | 111 | 24.5% (11.1, 43.0) |
|  |  | NUHY10 | Residents who have unplanned weight loss or are at risk of weight loss, received referral to a dietitian |  | U | Referral/ Consultation | 111 | 112 | 61.9% (42.9, 78.6) |
| 10 | Residents who have unplanned weight loss or are at risk of weight loss, and presenting with functional concerns, should receive referral to an occupational therapist. | NUHY11 | Residents who have unplanned weight loss or are at risk of weight loss, and are presenting with functional concerns, were referred to an occupational therapist. | (26) | U | Referral/ Consultation | 16 | 16 | nc |
| 11 | Residents who have unplanned weight loss or are at risk of weight loss or poor nutrition, should receive a medication review. | NUHY12 | Residents who have unplanned weight loss or are at risk of weight loss or poor nutrition, received a medication review. | (26, 53, 54) | U | Monitoring/ Review | 109 | 109 | 7.0% (2.4, 15.4) |
| 12 | Residents who are underweight or obese should be offered a specialised nutritional care plan. | NUHY13 | Residents who are underweight or obese were offered a specialised nutritional care plan. | (50) | U | Treatment | 75 | 75 | 54.8% (35.5, 73.1) |
| 13 | Residents who are underweight or obese who have a specialised nutritional care plan should have the following included:  - Goals  - Nutrition status - Address nutrition and hydration needs | NUHY14 | Residents who are underweight or obese who have a specialised nutritional\ care plan had the following included:    - Goals  - Nutrition\ status  - Address nutrition and hydration needs | (50) | U | Treatment | 41 | 41 | 62.6% (37.5, 83.5) |
| 14 | Residents who are underweight or obese who have a specialised nutritional care plan should have it reviewed by a dietitian monthly. | NUHY15 | Residents who are underweight or obese who have a specialised nutritional care plan had it reviewed by a dietitian monthly. | (50) | U | Monitoring/ Review | 41 | 42 | 6.6% (1.2, 18.7) |
| 15 | Residents who are underweight or obese and have a weight management program should receive quarterly monitoring by a dietitian. | NUHY16 | Residents who are underweight or obese and have a weight management program received quarterly monitoring by a dietitian. | (26, 51) | U | Monitoring/ Review | 43 | 43 | 28.5% (11.7, 51.2) |
| 16 | Residents^1^ who receive nutrition support should be provided with written information about their diagnosis and treatment options. | NUHY17 | Residents^1^ who receive nutrition support were provided with written information about their diagnosis and treatment options. | (54) | U | Information provision | 110 | 110 | 1.0% (0.0, 5.2) |
| 17 | Residents who receive nutrition support should be reviewed regularly. | NUHY18 | Residents who receive nutrition support were reviewed regularly. | (54) | U | Monitoring/ Review | 111 | 112 | 45.0% (28.8, 62.1) |
| 18 | Residents who have symptoms of dehydration should receive a hydration assessment | NUHY19 | Residents who have symptoms of dehydration received a hydration assessment | (117) | U | Diagnosis/ Assessment | 10 | 10 | nc |
| 19 | Residents who have symptoms of dehydration should be monitored daily. | NUHY20 | Residents who have symptoms of dehydration were monitored daily. | (117) | U | Monitoring/ Review | 10 | 10 | nc |
| 20 | Residents who have severe symptoms of dehydration or mild symptoms which do not improve within 24 hours of treatment, should receive: - a referral to a GP AND - treatment plan implemented AND  - daily fluid intake goals reviewed. | NUHY21 | Residents who have severe symptoms of dehydration or mild symptoms which do not improve within 24 hours of treatment, received a referral to a GP | (117) | U | Referral/ Consultation | 5 | 5 | nc |
|  |  | NUHY22 | Residents who have severe symptoms of dehydration or mild symptoms which do not improve within 24 hours of treatment, received a treatment plan implemented |  | U | Treatment | 4 | 4 | nc |
|  |  | NUHY23 | Residents who have severe symptoms of dehydration or mild symptoms which do not improve within 24 hours of treatment, received daily fluid intake goals reviewed. |  | U | Treatment | 3 | 3 | nc |
| **Oral and Dental Care** | | | | | | | | | |
| 1 | Residents should have a current oral health care plan. | ORAL01 | Residents had a current oral health care plan. | (50, 56-60, 118) | U | Treatment | 263 | 263 | 98.1% (95.6, 99.4) |
| 2 | Residents who have dentures should have their dentures disinfected once a week. | ORAL02 | Residents who have dentures had their dentures disinfected once a week. | (50) | U | Treatment | 148 | 148 | 12.4% (4.3, 26.3) |
| 3 | Residents who have dry mouth or lips should: - use a saliva substitute or oral lubricant or water-based lip balm and - drink water after meals, snacks and medication. | ORAL03 | Residents who have dry mouth or lips: - used a saliva substitute or oral lubricant or water-based lip balm and - were advised to drink water after meals, snacks and medication. | (50, 56) | U | Treatment | 22 | 22 | nc |
| 4 | Residents who have unexpected findings during oral care should be referred to their GP or dental professional. | ORAL04 | Residents who have unexpected findings during oral care were referred to their GP or dental professional. | (50, 56) | U | Referral/ Consultation | 21 | 21 | nc |
| 5 | Residents who have signs of oral disease or dysfunction that impact on their general health and well-being or have ill-fitting dentures, should be referred to their dental professional. | ORAL05 | Residents who have signs of oral disease or dysfunction that impact on their general health and well-being or have ill-fitting dentures, were referred to their dental professional. | (50, 56, 59) | U | Referral/ Consultation | 20 | 20 | nc |
| 6 | Residents who have medication side effects impacting on their oral health, should be referred for a Residential Medication Management Review (RMMR). | ORAL06 | Residents who have medication side effects impacting on their oral health, were referred for a Residential Medication Management Review (RMMR). | (56) | U | Referral/ Consultation | 3 | 3 | nc |
| 7 | Residents who have a functional or cognitive dysfunction that impacts on their ability to perform oral health tasks should be referred to the appropriate health service provider. | ORAL07 | Residents who have a functional or cognitive dysfunction that impacts on their ability to perform oral health tasks were referred to the appropriate health service provider. | (50, 119) | U | Referral/ Consultation | 12 | 12 | nc |
| 8 | Residents with hyposalivation or dry mouth should be offered appropriate prevention and treatment advice within 24 hours of diagnosis. | ORAL08 | Residents with hyposalivation or dry mouth were offered appropriate prevention and treatment advice within 24 hours of diagnosis. | (59) | U | Treatment | 6 | 6 | nc |
| 9 | Residents who have a change in the condition of their mouth or teeth should have an oral health assessment. | ORAL09 | Residents who have a change in the condition of their mouth or teeth had an oral health assessment. | (56) | U | Diagnosis/ Assessment | 22 | 22 | nc |
| **Pain** | | | | | | | | | |
| 1 | Residents should be asked about the current presence of pain: - when their condition significantly changes - any time pain is suspected - at least every three months - during scheduled assessments~~.~~ | PAIN01 | Residents who have a significant change in condition were asked about the current presence of pain | (34, 39, 40, 120, 121) | U | Diagnosis/ Assessment | 142 | 269 | 76.9% (66.0, 85.7) |
|  |  | PAIN02 | Residents for whom pain is suspected were asked about the current presence of pain |  | U | Diagnosis/ Assessment | 126 | 223 | 89.7% (80.2, 95.7) |
|  |  | PAIN03 | Residents were asked about the current presence of pain every 3 months |  | U | Diagnosis/ Assessment | 217 | 226 | 53.9% (41.4, 66.1) |
|  |  | PAIN04 | Residents were asked about the current presence of pain during scheduled assessments |  | U | Diagnosis/ Assessment | 3 | 3 | nc |
| 2 | Residents should receive a pain assessment using: - self-report AND/OR - observational (e.g., non-verbal or behavioural) | PAIN05 | Residents received a pain assessment using: - self-report AND/OR - observational (e.g., non-verbal or behavioural) | (39, 120, 121) | U | Diagnosis/ Assessment | 154 | 256 | 80.7% (70.8, 88.4) |
| 3 | Residents who have pain should be asked to describe the following characteristics of their pain: - quality AND - severity/intensity AND - duration/frequency and fluctuations throughout the day AND - pattern AND - site/location AND  - radiation/referral AND  - onset~~.~~ | PAIN06 | Residents who have pain were asked to describe the following characteristic of their pain: quality | (39, 120) | U | Diagnosis/ Assessment | 62 | 131 | 29.3% (16.5, 45.1) |
|  |  | PAIN07 | Residents who have pain were asked to describe the following characteristic of their pain: severity/intensity |  | U | Diagnosis/ Assessment | 62 | 131 | 58.4% (43.2, 72.6) |
|  |  | PAIN08 | Residents who have pain were asked to describe the following characteristic of their pain: duration/frequency and fluctuations throughout the day |  | U | Diagnosis/ Assessment | 63 | 132 | 16.8% (8.7, 28.1) |
|  |  | PAIN09 | Residents who have pain were asked to describe the following characteristic of their pain: pattern |  | U | Diagnosis/ Assessment | 62 | 131 | 9.8% (5.0, 16.9) |
|  |  | PAIN10 | Residents who have pain were asked to describe the following characteristic of their pain: site/location |  | U | Diagnosis/ Assessment | 63 | 133 | 86.4% (68.7, 96.2) |
|  |  | PAIN11 | Residents who have pain were asked to describe the following characteristic of their pain: radiation/referral |  | U | Diagnosis/ Assessment | 62 | 131 | 5.9% (2.3, 12.2) |
|  |  | PAIN12 | Residents who have pain were asked to describe the following characteristic of their pain: onset |  | U | Diagnosis/ Assessment | 62 | 131 | 23.3% (14.3, 34.4) |
| 4 | Residents who are unable to verbally report pain receive the following assessments: - locate pain AND - observation of pain behaviours and body language AND - identification of pathology that may be causing pain AND - obtaining a pain history from family or carers AND - response to pain relieving interventions. | PAIN13 | Residents who are unable to verbally report pain received the following assessment: locate pain | (39, 120, 122) | U | Diagnosis/ Assessment | 48 | 73 | 74.7% (42.9, 94.2) |
|  |  | PAIN14 | Residents who are unable to verbally report pain received the following assessment: observation of pain behaviours and body language |  | U | Diagnosis/ Assessment | 47 | 73 | 80.9% (57.1, 94.8) |
|  |  | PAIN15 | Residents who are unable to verbally report pain received the following assessments: identification of pathology that may be causing pain |  | U | Diagnosis/ Assessment | 46 | 70 | 56.8% (29.9, 81.0) |
|  |  | PAIN16 | Residents who are unable to verbally report pain received the following assessments: obtaining a pain history from family or carers |  | U | Diagnosis/ Assessment | 42 | 60 | 23.0% (4.3, 56.0) |
|  |  | PAIN17 | Residents who are unable to verbally report pain received the following assessments: response to pain relieving interventions |  | U | Diagnosis/ Assessment | 46 | 71 | 78.8% (51.8, 94.7) |
| 5 | Residents who have pain should receive a physical assessment. | PAIN18 | Residents who have pain received a physical assessment | (39, 120-123) (34) | U | Diagnosis/ Assessment | 102 | 162 | 32.0% (18.0, 48.9) |
| 6 | Residents who have pain should receive a functional assessment of their pain which includes: - activities of daily living (ADLs) and functional ability  - sleep and appetite. | PAIN19 | Residents who have pain received a functional assessment of their pain which includes: - activities of daily living (ADLs) and functional ability - sleep and appetite. | (34, 39, 120) | U | Diagnosis/ Assessment | 103 | 160 | 35.3% (20.6, 52.4) |
| 7 | Residents who have new pain should receive a psychosocial assessment of their pain. | PAIN20 | Residents who have new pain received a psychosocial assessment of their pain. | (39, 120, 122, 123) (34) | U | Diagnosis/ Assessment | 85 | 138 | 11.1% (2.1, 30.2) |
| 8 | Residents who have pain should have the effectiveness of their current treatments for pain evaluated. | PAIN21 | Residents who have pain had the effectiveness of their current treatments for pain evaluated. | (39, 40, 96, 120, 122, 124) | U | Treatment | 112 | 162 | 59.4% (44.4, 73.2) |
| 9 | Residents who have pain and whose pain relief is not adequate receive a consultation from a RN or Medical Practitioner within 24 hours. | PAIN22 | Residents who have pain and whose pain relief is not adequate received a consultation from a RN or Medical Practitioner within 24 hours. | (96, 120) | U | Referral/ Consultation | 47 | 54 | 86.2% (71.6, 95.1) |
| 10 | Residents presenting with acute pain of moderate to severe intensity or who appear to be in significant distress receive an assessment of: - their level of consciousness (LOC) including orientation to person/self, time and location - the characteristics of the pain  - their vital signs~~.~~ | PAIN23 | Residents presenting with acute pain of moderate to severe intensity or who appear to be in significant distress received an assessment of their level of consciousness (LOC) including orientation to person/self, time and location | (120) | U | Diagnosis/ Assessment | 74 | 116 | 2.2% (0.3, 7.5) |
|  |  | PAIN24 | Residents presenting with acute pain of moderate to severe intensity or who appear to be in significant distress received an assessment of the characteristics of the pain. |  | U | Diagnosis/ Assessment | 77 | 119 | 30.8% (16.1, 49.0) |
|  |  | PAIN25 | Residents presenting with acute pain of moderate to severe intensity or who appear to be in significant distress received an assessment of their vital signs. |  | U | Diagnosis/ Assessment | 74 | 116 | 24.7% (11.5, 42.5) |
| 11 | Residents who have acute and/or moderate to severe pain should receive immediate treatment. | PAIN26 | Residents who have acute and/or moderate to severe pain received immediate treatment. | (39, 120) | U | Treatment | 88 | 152 | 67.5% (53.7, 79.4) |
| 12 | Residents who have acute pain should not receive long-acting opioid preparations for pain management | PAIN27 | Residents who have acute pain received long-acting opioid preparations for pain management | (120) | O | Treatment | 89 | 132 | 84.0% (66.4, 94.7) |
| 13 | Residents who have acute pain and are administered analgesia should have their treatment response monitored within the hour. | PAIN28 | Residents who have acute pain and are administered analgesia had their treatment response monitored within the hour. | (39) | U | Monitoring/ Review | 82 | 148 | 55.6% (41.0, 69.5) |
| 14 | Residents who have chronic pain should have a current comprehensive plan of care. | PAIN29 | Residents who have chronic pain had a current comprehensive plan of care. | (34, 39, 40, 120, 121) | U | Treatment | 177 | 178 | 74.8% (60.3, 86.2) |
| 15 | Residents who have chronic pain and a comprehensive individualised plan of care had it developed in partnership with:  - resident - family or substitute decision-maker - multidisciplinary team. | PAIN30 | Residents who have chronic pain and a comprehensive individualised/ plan of care had it developed in partnership with:  - resident - family or substitute decision-maker - multidisciplinary team. | (34, 39, 40, 120, 122) | U | Treatment; Resident/ Family engagement | 152 | 152 | 66.7% (54.3, 77.7) |
| 16 | Residents who have chronic pain and a change in the level of pain should receive a full medical assessment and review of their medications | PAIN31 | Residents who have chronic pain and a change in the level of pain received a full medical assessment and review of their medications | (40) | U | Diagnosis/ Assessment | 31 | 31 | 74.9% (49.5, 91.8) |
| 17 | Residents who have chronic pain have analgesia prescribed as a 'regular' medication. | PAIN32 | Residents who have chronic pain had analgesia prescribed as a 'regular' medication. | (39, 40, 122) | U | Treatment | 174 | 174 | 73.4% (57.4, 86.0) |
| 18 | Residents who have pain and refuse analgesia should have the reasons documented. | PAIN33 | Residents who have pain and refuse analgesia had the reasons documented. | (120) | U | Documentation | 32 | 37 | 64.5% (35.0, 87.6) |
| 19 | Residents prescribed paracetamol must have a daily dose that does not exceed 4gm. | PAIN34 | Residents prescribed paracetamol had a daily dose that does not exceed 4gm. | (120) | U | Treatment | 204 | 204 | 96.9% (93.4, 98.8) |
| 20 | Residents prescribed NSAIDs or COX-2 selective inhibitors should have an assessment of their gastrointestinal and cardiovascular risk factors. | PAIN35 | Residents prescribed NSAIDs or COX-2 selective inhibitors had an assessment of their gastrointestinal and cardiovascular risk factors. | (123, 124) | U | Diagnosis/ Assessment | 28 | 28 | 2.4% (0.0, 16.4) |
| 21 | Residents commencing opioid therapy should be educated and counselled regarding proper opioid use, including: - warning signs of misuse or addiction - common side effects (e.g., nausea, constipation, dry mouth). | PAIN36 | Residents commencing opioid therapy were educated and counselled regarding proper opioid use, including: - warning signs of misuse or addiction - common side effects (e.g., nausea, constipation, dry mouth). | (96, 122, 123) | U | Information provision; Resident/ Family engagement | 40 | 40 | 10.5% (2.6, 25.7) |
| 22 | Residents receiving opioid therapy should be screened and monitored for opioid-related adverse effects. | PAIN37 | Residents receiving opioid therapy were screened and monitored for opioid-related adverse effects. | (39, 120, 122, 123) | U | Monitoring/ Review | 93 | 93 | 14.1% (2.5, 38.5) |
| 23 | Residents receiving opioid therapy should be prescribed laxative therapy. | PAIN38 | Residents receiving opioid therapy were prescribed laxative therapy. | (96, 97, 124) | U | Treatment | 98 | 98 | 90.2% (81.0, 96.0) |
| 24 | Residents^1^ who have chronic or long-term pain should receive education and information about their pain. | PAIN39 | Residents^1^ who have chronic or long-term pain received education and information about their pain. | (39, 120-122) | U | Information provision; Resident/ Family engagement | 152 | 152 | 2.1% (0.2, 8.3) |
| 25 | Residents who have inadequately managed pain should be referred to specialist services within 4 weeks. | PAIN40 | Residents who have inadequately managed pain were referred to specialist services within 4 weeks. | (40, 96) | U | Referral/ Consultation | 9 | 9 | nc |
| **Restraint** | | | | | | | | | |
| 1 | Residents who are being physically restrained had a multidimensional assessment prior to restraint use. | REST01 | Residents who are being physically restrained had a cognitive assessment prior to restraint use | (85) | U | Diagnosis/ Assessment | NA | NA | NA |
|  |  | REST02 | Residents who are being physically restrained had a medical history taken prior to restraint use |  | U | Diagnosis/ Assessment | NA | NA | NA |
|  |  | REST03 | Residents who are being physically restrained had a history of responsive behaviours documented prior to restraint use |  | U | Diagnosis/ Assessment | NA | NA | NA |
|  |  | REST04 | Residents who are being physically restrained had an assessment of the residents’ usual routines and preferences prior to restraint use |  | U | Diagnosis/ Assessment | NA | NA | NA |
|  |  | REST05 | Residents who are being physically restrained had a physical assessment prior to restraint use |  | U | Diagnosis/ Assessment | NA | NA | NA |
|  |  | REST06 | Residents who are being physically restrained had a pain assessment prior to restraint use |  | U | Diagnosis/ Assessment | NA | NA | NA |
|  |  | REST07 | Residents who are being physically restrained had an assessment of the resident’s communication ability prior to restraint use |  | U | Diagnosis/ Assessment | NA | NA | NA |
|  |  | REST08 | Residents who are being physically restrained had a screen for delirium prior to restraint use |  | U | Diagnosis/ Assessment | NA | NA | NA |
|  |  | REST09 | Residents who are being physically restrained had a screen for medicines that influence cognition prior to restraint use |  | U | Diagnosis/ Assessment | NA | NA | NA |
|  |  | REST10 | Residents who are being physically restrained had a mental state (mood disorders, psychosis) prior to restraint use |  | U | Diagnosis/ Assessment | NA | NA | NA |
|  |  | REST11 | Residents who are being physically restrained had a falls risk assessment prior to restraint use |  | U | Diagnosis/ Assessment | NA | NA | NA |
|  |  | REST12 | Residents who are being physically restrained had an assessment of the residents psychological coping strategies, cultural needs, level of stimulation etc prior to restraint use |  | U | Diagnosis/ Assessment | NA | NA | NA |
|  |  | REST13 | Residents who are being physically restrained had an assessment of the resident’s physical environment (noise, lighting) prior to restraint use |  | U | Diagnosis/ Assessment | NA | NA | NA |
|  |  | REST14 | Residents who are being physically restrained had a detailed history specific to behaviour triggers, how they present and what reduces them prior to restraint use |  | U | Diagnosis/ Assessment | NA | NA | NA |
|  |  | REST15 | Residents who are being physically restrained had the frequency, severity and level of distress of responsive behaviours documented prior to restraint use |  | U | Diagnosis/ Assessment | NA | NA | NA |
|  |  | REST16 | Residents who are being physically restrained had the level of risk associated with responsive behaviours documented prior to restraint use |  | U | Diagnosis/ Assessment | NA | NA | NA |
|  |  | REST17 | Residents who are being physically restrained had an individualised care plan for reducing responsive behaviours developed and implemented prior to restraint use |  | U | Treatment | NA | NA | NA |
|  |  | REST18 | Residents who are being physically restrained had their family (and/or substitute decision maker) informed of the risks of restraining and not restraining prior to restraint use |  | U | Diagnosis/ Assessment | NA | NA | NA |
| 2 | Residents who are being physically restrained should have Daily evaluation of behaviour and behaviour interventions. | REST19 | Residents who are being physically restrained should have daily evaluation of behaviour and behaviour interventions. | (85) | U | Monitoring/ Review | NA | NA | NA |
| **Skin Integrity** | | | | | | | | | |
| 1 | Residents should receive a skin wound risk assessment: - whenever the resident's condition significantly changes - monthly. | SKIN01 | Residents with a significant change in condition received a skin wound risk assessment. | (43-49) | U | Diagnosis/ Assessment | 149 | 228 | 18.5% (11.0, 28.3) |
|  |  | SKIN02 | Residents received a skin wound risk assessment monthly. |  | U | Treatment | 279 | 281 | 6.7% (3.7, 10.9) |
| 2 | Residents should receive a skin wound risk assessment which includes: - use of a validated risk assessment tool (e.g., for pressure injury: Braden scale, Norton risk-assessment scale, Waterlow score) | SKIN03 | Residents received a skin wound risk assessment including use of a validated risk assessment tool (e.g., for pressure injury: Braden scale, Norton risk-assessment scale, Waterlow score) | (43, 44, 46-49, 125, 126) | U | Diagnosis/ Assessment | 278 | 278 | 98.1% (92.2, 99.8) |
| 3 | Residents who are at medium/high risk of or have a skin wound should receive a care plan. | SKIN04 | Residents who are at medium/high risk of or have a skin wound received a care plan. | (27, 34, 43, 44, 48, 49, 125) | U | Treatment | 227 | 227 | 92.8% (81.6, 98.2) |
| 4 | Residents who have a skin wound should have the cause (when, where and how) assessed. | SKIN05 | Residents who have a skin wound had the cause (when, where and how) assessed. | (49) | U | Diagnosis/ Assessment | 149 | 215 | 70.6% (57.2, 81.8) |
| 5 | Residents who have a skin wound should have a current pain management plan. | SKIN06 | Residents who have a skin wound had a current pain management plan. | (44, 45, 49) | U | Treatment | 145 | 188 | 20.7% (10.5, 34.7) |
| 6 | Residents who have a skin tear should have a dressing applied and secured with non-adhesive silicone-interfaced dressing materials (such as arm/leg protectors, tubular wraps or flexible netting to further protect the skin). | SKIN07 | Residents who have a skin tear had a dressing applied and secured with non-adhesive silicone-interfaced dressing materials (such as arm/leg protectors, tubular wraps or flexible netting to further protect the skin). | (49) | U | Treatment | 64 | 79 | 11.4% (2.8, 28.1) |
| 7 | Residents who have a dressing on a skin tear should have the dressing marked with the date for removal and an arrow to indicate the direction for dressing removal. | SKIN08 | Residents who have a dressing on a skin tear had the dressing marked with the date for removal and an arrow to indicate the direction for dressing removal. | (49) | U | Diagnosis/ Assessment | 65 | 82 | 0.0% (0.0, 4.4) |
| 8 | Residents who have a skin tear and oedema and/or haematoma should be managed by: - applying gentle compression  - elevating the limb. | SKIN09 | Residents who have a skin tear and oedema and/or haematoma were managed by applying gentle compression | (49) | U | Treatment | 7 | 9 | nc |
|  |  | SKIN10 | Residents who have a skin tear and oedema and/or haematoma were managed by elevating the limb. |  | U | Treatment | 7 | 9 | nc |
| 9 | Residents^1^ who are at risk of pressure injuries should be provided with information on cause, prevention and treatment of pressure injuries. | SKIN11 | Residents^1^ who are at risk of pressure injuries were provided with information on cause, prevention and treatment of pressure injuries. | (44, 46, 48, 125) | U | Information provision | 212 | 212 | 2.0% (0.2, 6.8) |
| 10 | Residents who are at risk of pressure injuries should have a repositioning protocol including: - specifications about posture  - advising changing position at least every 6 hours if at risk or every 4 hours if high risk. | SKIN12 | Residents who are at risk of pressure injuries had a repositioning protocol including: - specifications about posture - advising changing position at least every 7 hours if at risk or every 4 hours if high risk. | (46-48) | U | Treatment | 218 | 218 | 74.8% (62.3, 84.9) |
| 11 | Residents who are at risk of or have a pressure injury should use pressure relieving/redistributing devices | SKIN13 | Residents who are at risk of or have a pressure injury had pressure relieving/redistributing devices used. | (45-48, 126, 127) | U | Monitoring/ Review | 218 | 218 | 73.8% (61.7, 83.7) |
| 12 | Residents who have a pressure injury should receive a mobility and support surface assessment on initial examination and whenever there is a significant change in the person's medical condition, weight, equipment, mobility, and/or pressure injury healing. | SKIN14 | Residents who have a pressure injury received a mobility and support surface assessment on initial examination | (45) | U | Treatment | 44 | 49 | 44.1% (14.8, 76.9) |
|  |  | SKIN15 | Residents who have a pressure injury and a significant change in the person's medical condition, weight, equipment, mobility, and/or pressure injury healing, received a mobility and support surface assessment. |  | U | Diagnosis/ Assessment | 33 | 33 | 27.4% (5.8, 62.1) |
| 13 | Residents who have a pressure injury should receive:  - prevention strategies (to prevent worsening) implemented within 8 hours AND - pressure injury assessment and pain assessment conducted AND  - a care plan updated or reviewed. | SKIN16 | Residents who have a pressure injury received prevention strategies (to prevent worsening) implemented within 8 hours | (43) | U | Treatment | 42 | 49 | 76.7% (49.4, 93.6) |
|  |  | SKIN17 | Residents who have a pressure injury received pressure injury assessment and pain assessment conducted |  | U | Treatment | 40 | 47 | 53.4% (25.2, 80.1) |
|  |  | SKIN18 | Residents who have a pressure injury received a care plan updated or reviewed. |  | U | Treatment | 42 | 47 | 36.4% (13.5, 65.0) |
| 14 | Residents who have a pressure injury should be repositioned at least every 4 hours. | SKIN19 | Residents who have a pressure injury were repositioned at least every 4 hours. | (45) | U | Treatment | 41 | 41 | 66.6% (21.8, 95.8) |
| 15 | Residents who have a pressure injury involving broken skin (i.e., grade 2 or more) should receive hydrocolloid or foam dressings | SKIN20 | Residents who have a pressure injury involving broken skin (i.e., grade 2 or more) received hydrocolloid or foam dressings | (128) | U | Treatment | 39 | 41 | 75.2% (52.5, 90.8) |
| 16 | Residents who have a pressure injury which is not healing at the optimal rate should receive a review of their wound management plan. | SKIN21 | Residents who have a pressure injury which is not healing at the optimal rate received a review of their wound management plan. | (43) | U | Diagnosis/ Assessment | 14 | 15 | nc |
| 17 | Residents who have non-healing wounds should be assessed for infection or biofilm. | SKIN22 | Residents who have non-healing wounds were assessed for infection or biofilm. | (125) | U | Diagnosis/ Assessment | 17 | 18 | nc |
| **Sleep** | | | | | | | | | |
| 1 | Residents who present with signs and symptoms of insomnia or have a change in symptoms should receive a comprehensive sleep assessment of at least 3 of the following: - sleep changes  - comorbidities - drug use - psychosocial - behavioural (habits and routines) - environmental (review of sleep environment) | SLEP01 | Residents who present with signs and symptoms of insomnia or have a change in symptoms received a comprehensive sleep assessment of at least 3 of the following: - sleep changes - comorbidities - drug use - psychosocial - behavioural (habits and routines) - environmental (review of sleep environment) | (67) | U | Diagnosis/ Assessment | 24 | 25 | 18.3% (2.9, 49.1) |
| 2 | Residents newly diagnosed with insomnia should have their medications reviewed within one week. | SLEP02 | Residents newly diagnosed with insomnia had their medications reviewed within one week. | (67) | U | Monitoring/ Review | 2 | 2 | nc |
| 3 | Residents who have newly diagnosed insomnia should receive non-pharmacological interventions as a first line treatment. | SLEP03 | Residents who have newly diagnosed insomnia received non-pharmacological interventions as a first line treatment. | (67) | U | Treatment | 3 | 3 | nc |
| 4 | Residents who have insomnia and are prescribed hypnotic drugs or melatonin should receive it for no more than four weeks at a time and at the lowest possible dose. | SLEP04 | Residents who have insomnia and are prescribed hypnotic drugs or melatonin received it for no more than four weeks at a time and at the lowest possible dose. | (67) | U | Treatment | 21 | 21 | nc |
| 5 | Residents who have insomnia and are prescribed a benzodiazepine class of hypnotic should receive fall prevention strategies. | SLEP05 | Residents who have insomnia and are prescribed a benzodiazepine class of hypnotic received fall prevention strategies. | (67) | U | Treatment | 19 | 19 | nc |
| 6 | Residents who have insomnia and are prescribed pharmacological interventions should be monitored. | SLEP06 | Residents who have insomnia and are prescribed pharmacological interventions were monitored. | (129) | U | Monitoring/ Review | 23 | 23 | nc |

LEGEND: nc=adherence not calculated; U= Underuse, O=Overuse;

1 and/or their family/substitute decision maker
