# Supplementary figures and images for: The quality of care delivered to residents in long-term care in Australia: an indicator-based review of resident records (CareTrack Aged study)
Source: BMC Med. 2024 Jan 23;22:22. doi: 10.1186/s12916-023-03224-8 (PMC10804560; doi:10.1186/s12916-023-03224-8)

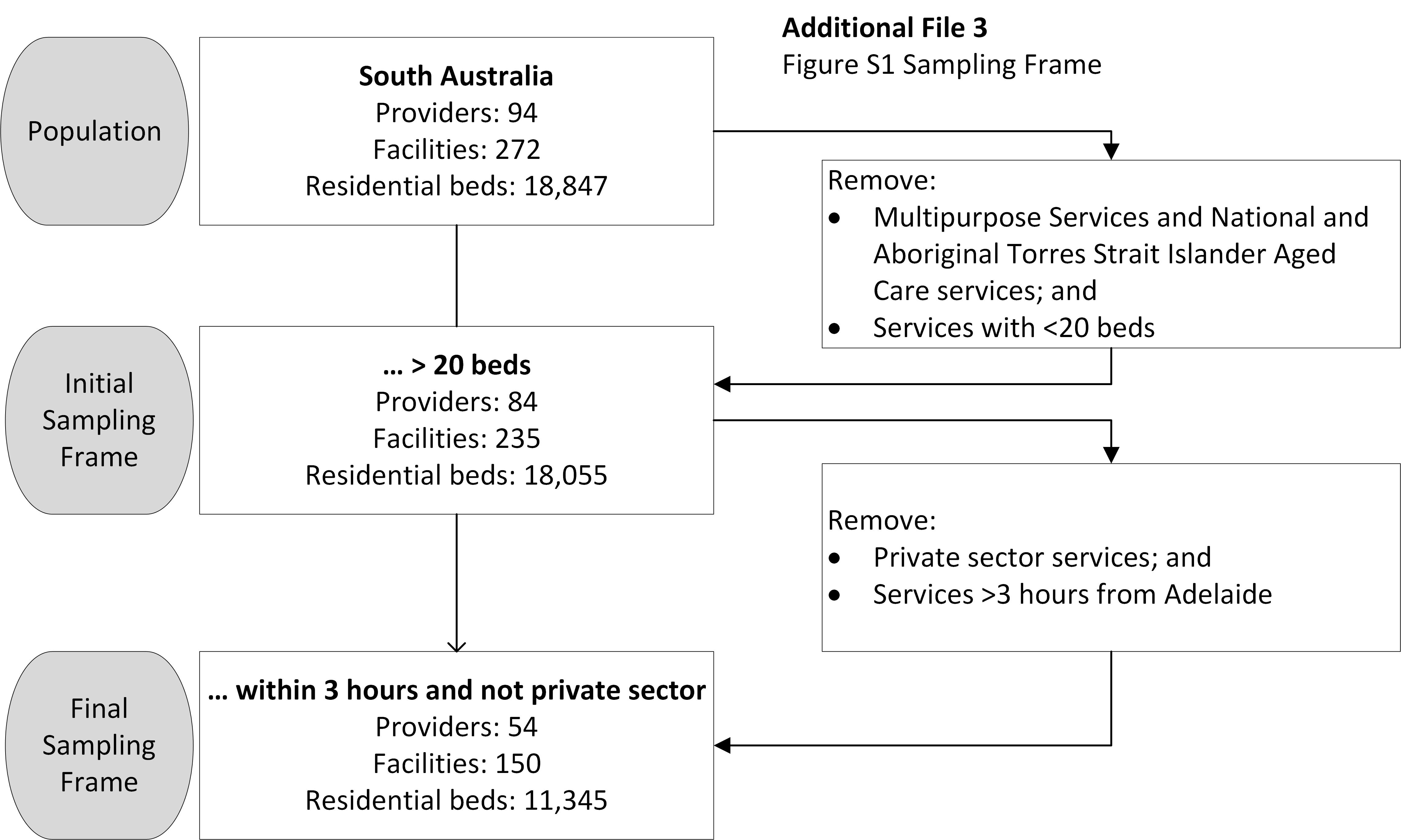

Supplement: Supplementary file 3 — Additional file 3: Figure S1. Sampling Frame. Sampling Frame flow diagram. [file 12916_2023_3224_MOESM3_ESM.jpg]
