# Supplementary material for: The quality of care delivered to residents in long-term care in Australia: an indicator-based review of resident records (CareTrack Aged study)
Source: BMC Med. 2024 Jan 23;22:22. doi: 10.1186/s12916-023-03224-8 (PMC10804560; doi:10.1186/s12916-023-03224-8)
Supplement: Supplementary file 4 — Additional file 4: Additional information on sampling and weighting. Additional information on the sampling, weighting and statistical analysis of the data. [file 12916_2023_3224_MOESM4_ESM.docx]

# Additional File 4: Additional information on sampling and weighting

***Sampling***:

Residents live in facilities which are operated by providers of different types including private for profit (excluded in this study), charitable providers and government providers. The research team initially recruited providers who in turn selected facilities where participation was possible in the target timeframes. Nominated facilities provided lists of eligible residents from whom participation was sought, where required.

***Sampling strategy***:

The Intracluster Correlation Coefficient (ICC) was not known for this sampling method and outcome. The sampling strategy therefore maximized the number of facilities that could be handled within the limitations imposed by available staffing and COVID. Within this number of facilities, sample size modelling indicated that large numbers of participants per facility was not required if the ICC was assumed to be low, and only provided limited additional statistical power when the ICC was assumed to be high. Thus, a number of 12 residents per facility was selected, with oversampling of new admissions and recent deaths to improve the estimates of adherence for indicators only applicable to these groups.

***Weights***:

An overall sampling fraction was created for each participant type (admitted in target period, died in target period, resident throughout target period to address differential sampling by participant type, facility size, organization size, and organisation type. Participants who were admitted or who died during the audit period were substantially oversampled, so weights were created to return these to their estimated rates of natural occurrence. Each of 24 facilities identified the number of residents admitted and dying during the target period. A small number of residents who were admitted also died during the target period (3.9% of those admitted in the target period, or an estimated 0.3% of the study population); there were too few of these to reliably estimate, so this small group was removed from the study population. There were, thus, three ***components*** to the study population, defined by their status in the target period: 1) Residents who were neither admitted nor who died (fraction=81.3%); 2) Residents who were admitted (6.1%); and 3) Residents who died (6.0%). People in these groups thus represent an estimated 93.5% of the number of licensed residential beds in the 24 facilities that reported these numbers.

Weights were calculated using the formula below: An overall sampling fraction was created to address differential sampling by participant type, facility size, organization size, and organization type:

$$\boldsymbol{Sampling fraction(i)=Facility fraction(i) X Provider fraction(i) X Org. type fraction(i)}$$

Where *i* = Admitted during target period (Group A), Resident throughout the target period (Group B), or Died during the target period (Group C).

A ***within-facility sampling fraction*** was calculated within each of the three population components. For each group, this sampling fraction was based on reported numbers for the 24 facilities that provided this data and estimated numbers in the twenty-fifth facility. In one facility there were no admitted patients in the target period and in another there were no patients who died; in both circumstances, the sampling fractions for other facilities of the same provider were adjusted to ensure that the providers relative contributions were maintained.

A ***within-provider*** ***sampling fraction*** was calculated as the proportion of all approved residential places operated by the organization that were in sampled facilities. For ten of the 13 providers all facilities were sampled, and therefore this fraction was 1.0.

Sampling was not performed within pre-defined strata and, as a result, there was substantial variation in effective ***sampling fractions by organization type***. Licensed beds in sampled providers represented only 5.7% of (n=3872) beds operated by charitable organizations, 19.6% of (n=1537) beds run by community organizations, 44.5% of (n=5233) beds in facilities run by religious groups, 62.6% of (n=214) local government and 34.2% of (n=729) state government beds. For each organization type, a sampling fraction was created as the number of approved residential places in sampled providers as a proportion of the number of approved beds in that organization type.

Inverting the final sampling fraction creates weights which, when applied to sampled residents, approximates the total number of estimated residential beds in the study population that were estimated to be in the three target groups (i.e., 93.5% of funded beds). Because of inter-facility differences in the proportion admitted and died during the target period, there was substantial variation between the summed weights and the known number of beds by facility type. The weights were therefore re-weighted: i) using the known number of beds by organization n type; and ii) using the estimated number of residents by group (admitted/died/neither in the study period). After this ‘ranking’ procedure(133), the summed weights were within one percent of the estimated number of residents for each of the five organization types and the estimated number in each of the three study population components.

*Statistical analysis*:

Statistical analysis was performed using the SURVEYFREQ procedure in SAS 9.4. Estimates at all levels controlled for clustering at the level of the provider; this choice of cluster reflects the ultimate-cluster procedure as this controls for clustering of indicator assessments by facility and resident, which are in turn clustered by provider. Indicator-specific and condition-level estimates controlled for stratification by organization type, with charitable, local and state government organisations grouped as a single omnibus type, as each of these organization types had only one cluster (provider); this results in three pseudostrata – religious organizations, community-based organizatons and other organizations. The overall estimate additionally controlled for stratification by condition; this was also true for analyses by indicator type (overuse/underuse) and selected phases of care (Diagnosis/Assessment, Treatment and Monitoring/Review). Estimates of average adherence by facility controlled for clustering by patient and were not stratified. Estimates of average adherence by indicators did not control for clustering, but pseudostratified by organization type. All estimates were weighted using the weights derived using the methods described above.
