# Supplementary material for: The quality of care delivered to residents in long-term care in Australia: an indicator-based review of resident records (CareTrack Aged study)
Source: BMC Med. 2024 Jan 23;22:22. doi: 10.1186/s12916-023-03224-8 (PMC10804560; doi:10.1186/s12916-023-03224-8)
Supplement: Supplementary file 5 — Additional file 5: Table S3. ICCs overall and by condition. The weighted (unweighted for admission) ICCs overall and by condition. [file 12916_2023_3224_MOESM5_ESM.docx]

# Additional File 5: ICCs overall and by condition

**TableS3: ICCs overall and by condition**

| **Condition** | **ICC** |
| --- | --- |
| Admission^a^ | 0.027 |
| Bladder and Bowel | 0.179 |
| Cognitive Impairment | 0.106 |
| Depression | 0.542 |
| Dysphagia | 0.113 |
| End-of-life Care | 0.036 |
| Infection | 0.034 |
| Medication | 0.092 |
| Mobility and Falls | 0.046 |
| Nutrition and Hydration | 0.009 |
| Oral Health | 0.026 |
| Pain | 0.057 |
| Skin Integrity | 0.011 |
| Sleep | 0.045 |
| All | 0.023 |

a Unweighted – weighted version would not converge
